# Supplementary material for: Evaluation of the Antioxidant and Antimicrobial Activity of Natural Deep Eutectic Solvents (NADESs) Based on Primary and Specialized Plant Metabolites
Source: Molecules. 2025 Oct 29;30(21):4219. doi: 10.3390/molecules30214219 (PMC12609234; doi:10.3390/molecules30214219)

molecules

MDPI

Article

Evaluation of the Antioxidant and Antimicrobial Activity of Natural Deep Eutectic Solvents (NADESs) Based on Primary and Specialized Plant Metabolites

Magdalena Kulinowska [1], Agnieszka Grzegorczyk [2], Sławomir Dresler [1,3,*], Agnieszka Skalska-Kamińska [1], Katarzyna Dubaj [4] and Maciej Strzemski [1,*]

[1] Department of Analytical Chemistry, Medical University of Lublin, 20-093 Lublin, Poland; kulinowskamagdalena@gmail.com (M.K.); agnieszka.skalska-kaminska@umlub.edu.pl (A.S.-K.)
[2] Department of Pharmaceutical Microbiology, Medical University of Lublin, 20-093 Lublin, Poland; agnieszka.grzegorczyk@umlub.edu.pl
[3] Department of Plant Physiology and Biophysics, Institute of Biological Sciences, Faculty of Biology and Biotechnology, Maria Curie-Skłodowska University, 19 Akademicka St., 20-033 Lublin, Poland
[4] Department of Basic Medical Sciences, Faculty of Medical and Health Sciences, Casimir Pulaski Radom University, 27 Boleslawa Chrobrego Str., 26-600 Radom, Poland; k.dubaj@urad.edu.pl
* Correspondence: dresler.slawomir@gmail.com (S.D.); maciej.strzemski@umlub.edu.pl (M.S.)

 https://doi.org/10.3390/30214219

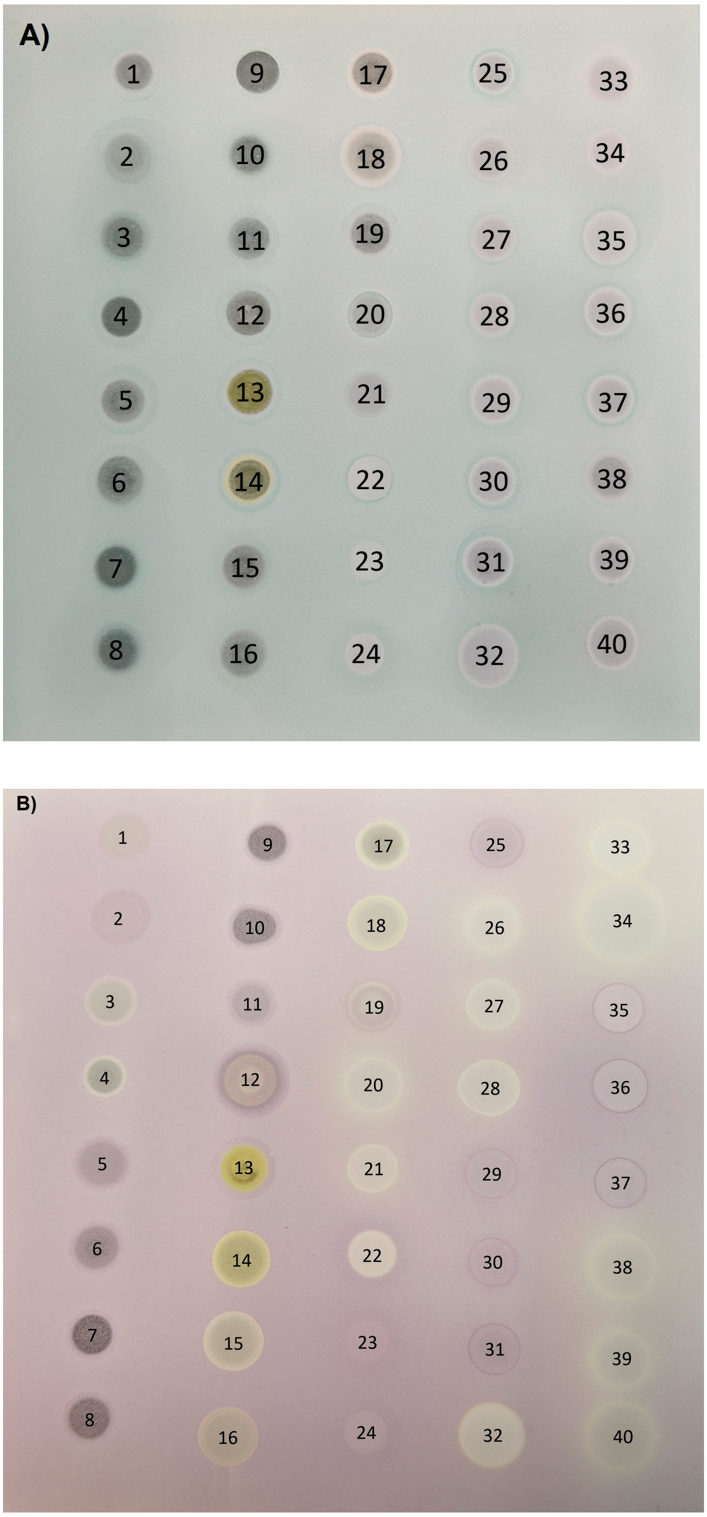

Figure S1. Photographs of bioautograms on HPTLC RP-18 plates after spraying with free radical solutions: A) - ABTS, B) – DPPH.

Table S1. Antioxidants' activity of NADESs tested. Values are expressed as trolox equivalent (mg Trolox/mL NADES); ND-not detected

No.	ABTS	DPPH	FRAP	CUPRAC
**Hydrophilic PRIM based - NADES**				
1	0.003±0.000 j	0.272±0.003 f	0.008±0.003 e	0.000±0.000 ND
2	0.007±0.004 j	0.144±0.005 f	0.000±0.000 ND	0.000±0.000 ND
3	0.011±0.001 j	0.255±0.012 f	0.000±0.000 ND	0.000±0.000 ND
4	0.004±0.001 j	0.347±0.018 f	0.000±0.000 ND	0.000±0.000 ND
5	0.002±0.001 j	0.035±0.008 f	0.000±0.000 ND	0.106±0.001 l
6	0.009±0.001 j	0.015±0.009 f	0.008±0.004 e	0.001±0.000 l
7	0.012±0.001 j	0.018±0.006 f	0.000±0.000 ND	0.000±0.000 ND
8	0.028±0.002 j	0.032±0.003 f	0.015±0.004 e	0.022±0.001 l
9	0.053±0.000 j	0.042±0.007 f	0.031±0.002 e	0.044±0.001 l
10	0.011±0.000 j	0.029±0.001 f	0.006±0.002 e	0.008±0.000 l
11	0.009±0.004 j	0.000±0.000 ND	0.000±0.000 ND	0.000±0.000 ND
**Hydrophilic HEVO based - NADES**				
12	0.372±0.075 j	0.303±0.038 f	0.634±0.034 e	0.862±0.049 l
13	16.364±27.107 hi	127.751±4.191 c	166.266±±20.147 c	199.824±5.883 i
14	68.242±7.511 ij	113.702±2.951 c	75.364±1.081 d	170.854±8.451 i
15	89.138±3.027 ij	0.340±0.012 f	0.701±0.054 e	0.907±0.058 l
16	0.316±0.001 j	0.260±0.013 f	4.745±0.340 e	0.116±0.005 l
17	395.092±60.464 g	155.070±2.703 b	330.247±37.555 b	813.880±20.058 a
18	521.677±86.287 fg	547.806±33.731 a	529.624±42.580 a	501.800±6.211 b
19	0.030±0.001 j	0.131±0.004 f	0.038±0.003 e	0.005±0.000 l
**Lipophilic HEVO based – NADES**				
20	861.246±33.029 a	36.648±0.846 d	23.577±1.000 e	468.240±19.368 bcd
21	617.453±77.246 def	32.005±1.093 de	11.354±0.691 e	472.390±19.720 bc
22	167.373±24.167 hi	13.943±0.780 ef	2.358±0.232 e	54.601±1.601 k
23	0.034±0.008 j	0.168±0.093 f	0.000±0.000 ND	0.368±0.012 l

24	0.047±0.010 j	0.176±0.093 f	0.000±0.000 ND	0.231±0.027 l
25	0.040±0.018 j	0.195±0.127 f	0.000±0.000 ND	0.097±0.003 l
26	729.972±52.575 abcd	35.360±0.702 de	17.143±0.977 e	415.950±20.331 ef
27	632.187±44.214 def	32.465±1.919 de	13.550±1.034 e	365.735±18.890 g
28	667.015±48.008 de	31.623±1.368 de	10.763±0.664 e	251.195±5.116 h
29	0.019±0.006 j	0.162±0.106 f	0.000±0.000 ND	0.082±0.007 l
30	0.049±0.003 j	0.178±0.092 f	0.000±0.000 ND	0.069±0.002 l
31	0.033±0.019 j	0.218±0.002 f	0.000±0.000 ND	0.074±0.002 l
32	234.349±71.893 h	16.590±4.300 def	4.714±1.096 e	128.355±3.521 j
33	558.514±80.477 ef	29.146±4.562 de	15.145±1.625 e	405.990±16.265 f
34	848.520±24.167 ab	34.871±1.653 de	19.033±1.460 e	452.678±17.789 cde
35	0.061±0.022 j	0.166±0.095 f	0.016±0.005 e	0.166±0.003 l
36	0.025±0.011 j	0.190±0.098 f	0.000±0.000 ND	0.403±0.007 l
37	0.032±0.008 j	0.172±0.086 f	0.000±0.000 ND	0.299±0.016 l
38	720.596±27.906 bcd	35.360±1.765 de	15.118±0.825 e	432.135±10.613 def
39	691.796±61.472 cde	35.975±0.505 de	17.654±1.029 e	334.195±18.781 g
40	806.325±17.362 abc	35.692±1.124 de	16.838±1.376 e	429.023±7.624 ef

molecules

MDPI

Table S2. Antimicrobial activity of tested NADESs. Values are expressed as a diameters of the zones of inhibition (mm).

	**Gram-positive bacteria**					**Gram-negative bacteria**			***Candida* Yeasts**		
	Staphylococcus epidermidis	*Bacillus cereus*	MSSA	MRSA	*Enterococcus faecalis*	*Salmonella* Tiphimurium	*Escherichia coli*	*Pseudomonas aeruginosa*	*C. albicans* ATCC 10231	*C. glabrata* ATCC 90030	*C. auris* CDC B11903
	Vancomycin (30µg)					Ciprofloxacin (5 µg)			Fluconazole (25 µg)		
	19.67±0.58<br>hij	12.67±3.21<br>klm	16.33±2.08<br>fghi	15.5±2.12<br>ij	11.00±2.83<br>kl	33.00±4.58<br>a	32.00±1.41<br>a	29.33±5.77<br>a	19.67±1.15<br>jk	18.67±1.15<br>l	23.33±0.58<br>hij
No.	**Hydrophilic PRIM based – NADES**										
1	14.33±2.08<br>mno	13.67±1.53<br>ijkl	10.67±1.15<br>op	12.33±1.15<br>lm	12.00±0.00<br>jk	10.00±0.00<br>jk	11±1.00<br>mno	12.33±1.15<br>fg	0.00±0.00<br>n	0.00±0.00<br>p	6.67±0.58<br>r
2	13.33±4.04<br>opq	13±1.00<br>jklm	7.33±1.15<br>q	8.17±0.76<br>no	11.33±0.58<br>klm	8.00± 0.00<br>o	8.33±0.58<br>rst	10.67±2.52<br>hi	6.67±0.58<br>m	7.00±1.00<br>o	8.33±0.58<br>p
3	19.00±2.65<br>ijk	16±1.00<br>gh	12.33±0.58<br>mno	12.33±0.58<br>lm	13.33±0.58<br>hij	8.33±0.58<br>no	11.00±1.00<br>mno	13.00±2.00<br>fg	0.00±0.00<br>n	0.00±0.00<br>p	0.00±0.00<br>s
4	23.67±0.58<br>fgh	18±1.00<br>fg	15±2.65<br>hijk	15.67±2.08<br>ij	18.33±3.06<br>efgh	10.33±2.08<br>jkl	13.33±1.53<br>jk	18.67±0.58<br>cd	0.00±0.00<br>n	0.00±0.00<br>p	6.67±0.58<br>r
5	6.33±0.58<br>t	6.67±1.15<br>r	0.00±0.00<br>r	6.67±1.15<br>p	6.33±0.58<br>q	6.33±0.58<br>p	7.00±0.00<br>st	6.67±0.58<br>o	0.00±0.00<br>n	0.00±0.00<br>p	6.67±0.58<br>r
6	6.33±0.58<br>t	6.67±0.58<br>qr	6.67±1.15<br>q	6.67±1.15<br>p	6.33±0.58<br>q	0.00±0.00<br>q	7.00±0.00<br>st	6.33±0.58<br>o	6.33±0.58<br>m	6.67±0.58<br>o	6.33±0.58<br>r
7	6.33±0.58<br>t	6.67±0.58<br>qr	6.67±1.15<br>q	6.67±1.15<br>p	0.00±0.00<br>r	0.00±0.00<br>q	0.00±0.00<br>u	0.00±0.00<br>p	0.00±0.00<br>n	0.00±0.00<br>p	6.33±0.58<br>r
8	0.00±0.00<br>u	0.00±0.00<br>s	6.67±1.15<br>q	0.00±0.00<br>q	0.00±0.00<br>r	0.00±0.00<br>q	0.00±0.00<br>u	0.00±0.00<br>p	0.00±0.00<br>n	0.00±0.00<br>p	6.67±0.58<br>r
9	0.00±0.00<br>u	6.33±0.58<br>r	6.67±0.58<br>q	6.33±0.58<br>p	0.00±0.00<br>r	6.67±0.58<br>p	0.00±0.00<br>u	0.00±0.00<br>p	0.00±0.00<br>n	0.00±0.00<br>p	6.67±0.58<br>r
10	0.00±0.00<br>u	0.00±0.00<br>s	6.67±0.58<br>q	6.33±0.58<br>p	0.00±0.00<br>r	6.67±0.58<br>p	0.00±0.00<br>u	0.00±0.00<br>p	0.00±0.00<br>n	0.00±0.00<br>p	6.33±0.58<br>r
11	0.00±0.00<br>u	6.33±0.58<br>r	6.67±0.58<br>q	0.00±0.00<br>q	6.33±0.58<br>q	6.67±0.58<br>p	6.67±0.58<br>t	0.00±0.00<br>p	0.00±0.00<br>n	6.67±0.58<br>o	0.00±0.00<br>s
	**Hydrophilic HEVO based - NADES**										

12	6.33±0.58 t	6.33±0.58 r	7.00±1.00 q	6.67±1.15 p	0.00±0.00 r	6.67±0.58 p	0.00±0.00 u	0.00±0.00 p	0.00±0.00 n	6.67±0.58 o	0.00±0.00 s
13	7.67±0.58 st	0.00±0.00 s	6.67±0.58 q	6.67±0.58 p	0.00±0.00 r	0.00±0.00 q	7.00±0.00 st	6.67±0.58 p	0.00±0.00 n	0.00±0.00 p	0.00±0.00 s
14	9.00±3.46 u	6.67±0.58 qr	0.00±0.00 r	6.67±0.58 p	0.00±0.00 r	6.67±0.58 p	0.00±0.00 u	6.33±0.58 o	0.00±0.00 n	0.00±0.00 p	0.00±0.00 s
15	11.00±1.00 rs	11±0.00 nop	10.33±0.58 p	10±0.00 mn	10.33±1.15 mno	9.00±0.00 lmn	9.00±0.00 qrs	10.33±0.58 i	0.00±0.00 n	0.00±0.00 p	6.67±0.58 r
16	14.00±0.00 no	11±1.00 no	14.33±0.58 ijkl	13.67±0.58 kl	11.00±1.00 lmn	12.33±0.58 hi	12.33±0.58 kl	12.33±1.15 fg	7.00±0.00 lm	7.00±0.00 o	7.33±0.58 q
17	11.67±1.15 qr	9±0.00 op	13.67±0.58 jklm	12.33±1.15 lm	10.00±1.00 no	9.33±0.58 klm	10.00±0.00 pqr	9.67±0.58 ij	6.67±0.58 m	7.00±1.00 o	6.33±0.58 r
18	13.00±0.00 opq	11±1.00 no	15.33±1.15 ghij	14±0.00 jk	13.00±2.00 ijk	11.00±1.00 ij	12.67±1.15 kl	11.67±0.58 gh	7.67±0.58 l	7.00±0.00 o	7.33±0.58 q
19	13.33±2.31 op	11.67±0.58 mn	11.33±0.58 nop	11.67±6.66 m	9.67±0.58 op	11.00±1.00 ij	11.33±0.58 mn	10.33±0.58 i	7.50±0.50 l	6.67±0.58 o	7.33±0.58 q
**Lipophilic HEVO based – NADES**											
20	44.67±2.52 a	34.67±0.58 a	33.67±3.06 a	39.33±2.52 a	28.00±1.00 ab	26.67±2.08 ab	26.67±0.58 ab	8.67±0.58 jk	65.33±1.15 a	59.67±2.52 a	72.33±2.08 ab
21	34.33±2.31 bcd	25.67±2.08 cd	23.00±1.73 bc	28.67±1.15 cde	21.67±0.58 cdef	18.00±2.00 ef	17.33±0.58 ghi	7.00±0.00 no	48.00±1.73 e	50.67±2.52 de	66.00±1.73 abc
22	18.67±2.52 ijk	17.67±1.53 fg	14.67±1.53 hijkl	19.67±3.51 gh	11.00±1.00 lmn	14.67±1.53 gh	11.67±1.53 lm	7.00±0.00 no	30.33±1.53 i	19.33±1.53 kl	20.00±2.65 jklm
23	7.00±0.00 t	8.33±1.53 pq	7.00±0.00 q	7.33±0.58 op	0.00±0.00 r	9.33±0.58 klm	11.33±0.58 mn	7.33±0.58 mn	10.00±0.00 k	11.33±0.58 n	9.67±0.58 op
24	11.67±0.58 qr	14.00±1.73 ijk	13.00±1.00 lmn	15.00±2.00 jk	8.67±0.58 pq	8.67±1.15 mno	11.00±1.00 mno	0.00±0.00 p	25.33±2.08 j	12.67±0.58 mn	13.67±0.58 nop
25	14.33±1.53 mno	15.00±1.73 hi	13.67±1.53 jklm	18.67±1.53 gh	10.33±1.15 mno	8.33±0.58 no	10.00±1.00 opq	0.00±0.00 p	40.33±2.52 f	30.33±2.89 i	41.00±3.00 fgh
26	33.00±1.73 de	28.67±3.79 bc	26.67±2.31 ab	32.00±1.73 bc	23.67±1.15 bcde	20.00±1.00 cde	22.33±2.31 bcdef	7.67±0.58 lm	50.67±2.08 de	50.67±3.21 de	60.67±2.52 cde
27	28.00±1.73 efg	20.33±0.58 def	19.67±0.58 cde	27.00±1.00 de	19.00±1.00 defg	13.00±1.00 gh	15.67±0.58 hij	0.00±0.00 p	40.00±1.00 f	40.00±2.00 fg	51.33±3.51 efg
28	22.67±1.53 ghi	18.33±1.53 efg	16.67±1.15 efgh	20.33±2.08 fg	15.33±1.15 ghi	12.33±1.15 hi	14.00±1.73 ijk	0.00±0.00 p	38.00±1.00 g	37.00±1.00 gh	54.67±2.08 ef

29	16.00±1.73 klm	14.67±2.08 hij	12.33±0.58 mno	15.33±1.53 ijk	11.67±0.58 kl	9.33±0.58 klm	11.00±1.73 mno	0.00±0.00 p	37.67±1.15 g	17.67±2.08 lm	20.00±1.00 jkl
30	14.00±1.00 nop	12.33±0.58 lmn	13.33±1.15 klm	17.00±1.00 hi	10.00±0.00 op	9.00±1.00 lmn	10.67±0.58 nop	0.00±0.00 p	34.00±1.73 h	17.67±1.53 l	17.33±0.58 lmn
31	12.67±0.58 pqr	13.67±1.53 ijkl	12.33±2.31 mnop	15.67±1.15 ij	10.33±0.58 no	8.67±0.58 mno	10.67±0.58 nop	0.00±0.00 p	36.67±2.52 g	32.00±3.46 hi	34.33±3.79 ghi
32	23.67±0.58 fgh	22.67±0.58 de	21.67±1.15 bcd	23.67±1.53 ef	18.33±0.58 fgh	20.33±1.53 cde	19.67±1.53 efg	23.67±1.15 ab	38.00±1.00 g	38.33±2.52 g	43.33±2.08 fgh
33	31.67±1.53 def	26.33±0.58 cd	26.67±1.53 ab	28.33±0.58 cde	23.67±2.31 bcd	18.33±1.53 ef	20.33±1.53 defg	19.00±1.00 bcd	53.33±0.58 bc	46.67±3.06 ef	59.00±2.00 de
34	38.67±0.58 ab	28.00±1.00 bc	30.33±2.89 a	34.33±1.53 ab	26.67±1.53 ab	20.67±0.58 abc	24.67±2.08 abc	14.67±0.58 de	59.00±1.73 ab	52.33±3.06 cd	64.00±2.00 bcd
35	16.67±1.15 jkl	19.00±0.00 ef	18.00±2.65 defg	20.33±2.08 fg	12.33±1.15 jk	19.00±1.00 def	19.33±0.58 fgh	26.67±0.58 a	30.33±0.58 i	23.00±1.00 jk	16.00±1.00 mno
36	14.00±0.00 no	16.33±0.58 gh	18.00±0.00 cdef	20.67±1.53 fg	11.67±0.58 kl	16.33±0.58 fg	15.33±1.53 hij	20.33±0.58 bc	30.67±1.53 i	26.00±3.46 ij	18.33±0.58 klm
37	14.67±0.58 lmn	14.00±1.00 ijk	17.00±1.00 efg	19.00±1.00 gh	9.67±0.58 op	12.33±1.53 hi	12.67±0.58 k	13.33±1.15 ef	39.33±1.15 f	26.67±1.53 ij	21.33±1.53 ijk
38	36.67±2.08 abcd	28.67±1.53 abc	31.67±2.08 a	36.00±1.73 ab	24.33±0.58 bc	20.33±0.58 bcd	22.67±1.53 bcd	8.33±0.58 kl	52.00±2.00 cd	53.00±1.00 cd	64.33±1.53 bcd
39	33.33±1.53 cde	30.00±2.65 abc	28.00±4.58 ab	31.33±2.89 bcd	26.67±1.53 ab	18.67±0.58 ef	21.67±0.58 cdef	7.67±0.58 lm	52.33±0.58 cd	54.67±2.08 bc	74.33±1.53 a
40	38.33±2.52 abc	30.33±0.58 ab	31.00±3.61 a	32.33±1.53 bc	30.33±2.52 a	20.33±0.58 bcd	22.33±1.15 bcde	8.33±0.58 kl	51.00±1.00 d	56.67±1.53 ab	69.33±7.02 ab

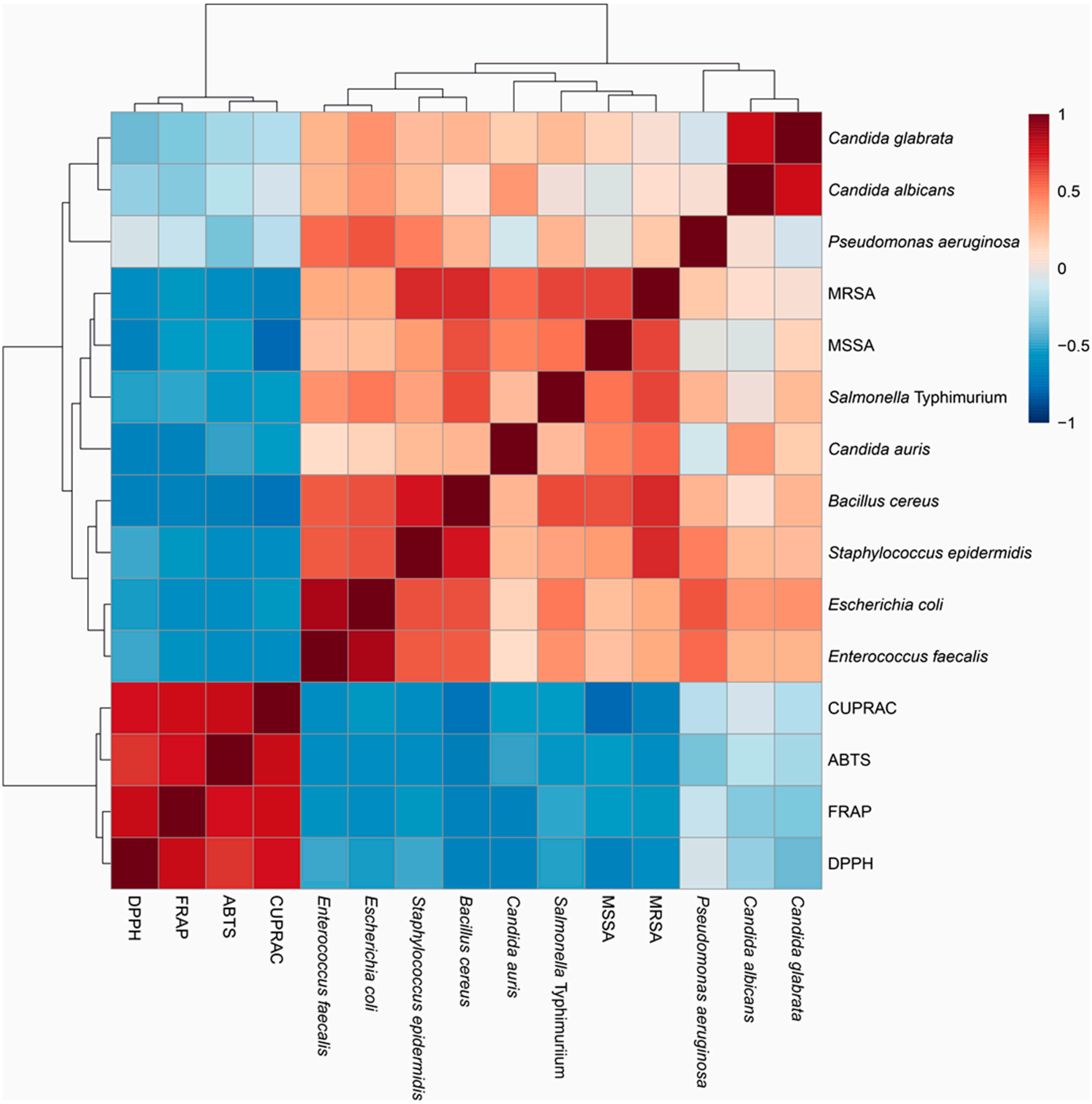

Figure S2. Heatmap of Pearson correlation coefficients (red = positive, blue = negative) with hierarchical clustering using Euclidean distance and Ward's method.

Table S3. Graphical representation of the results from the halo assay (disk diffusion test).

Staphylococcus epidermidis ATCC 12228

Hydrophilic NADES with positive-control

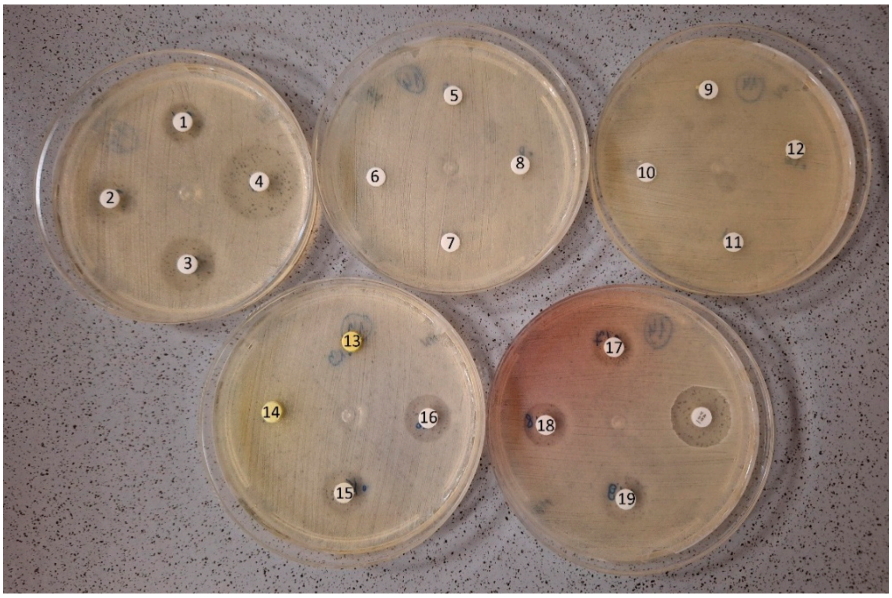

Lipophilic NADES

1,8-Cineole:Thymol

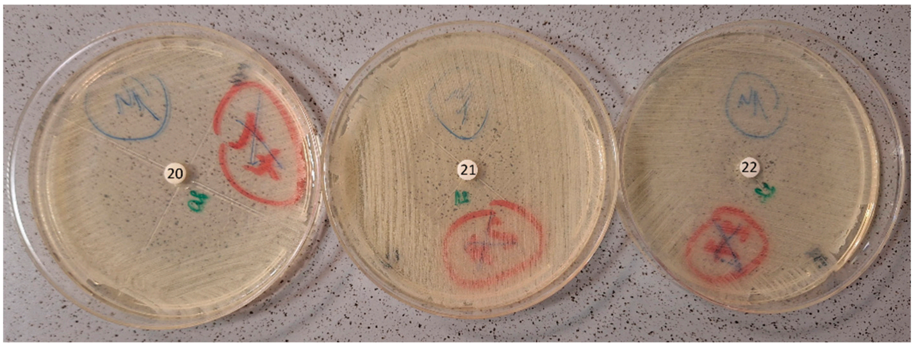

***1,8*-Cineole:DL-Menthol**

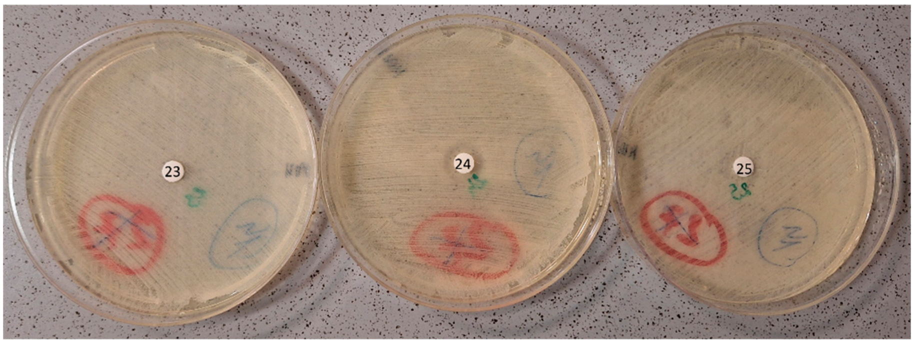

Camphor:Thymol

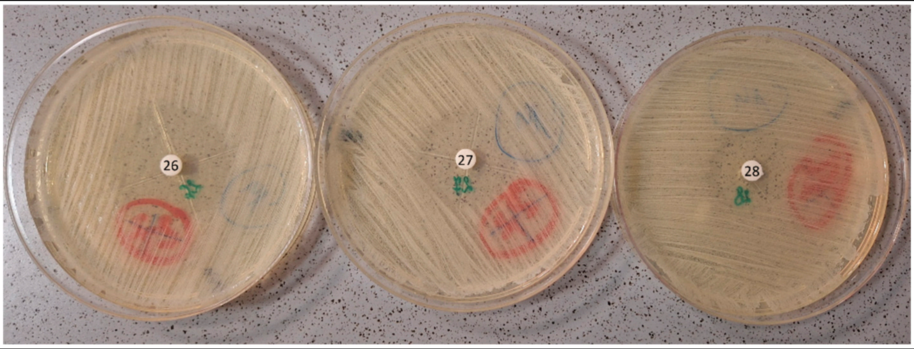

Camphor:DL-Menthol

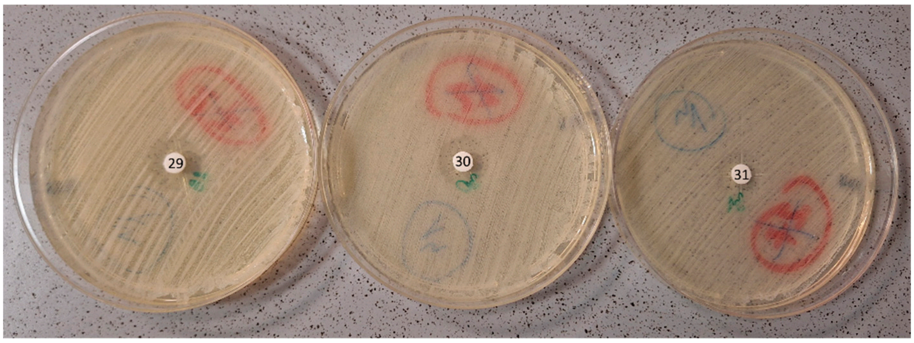

Benzyl alcohol:Thymol

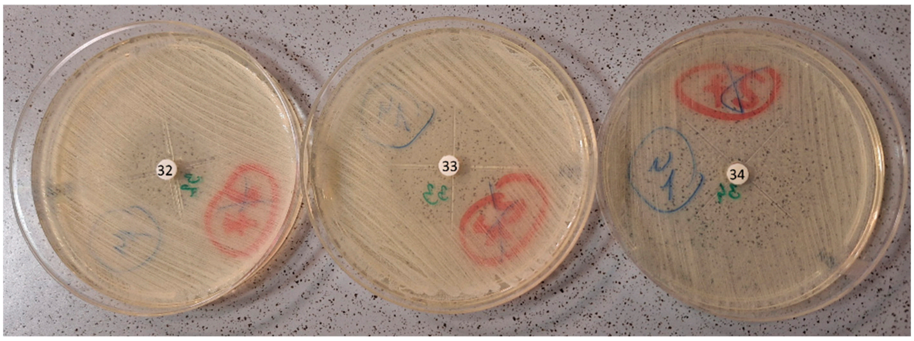

Benzyl alcohol:DL-Menthol

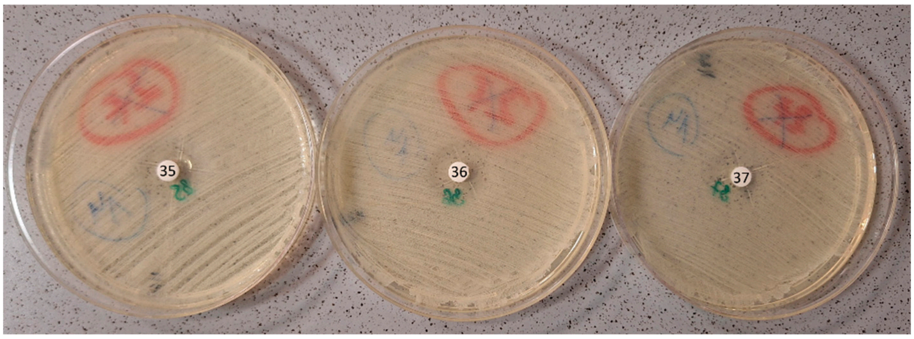

Borneol:Thymol

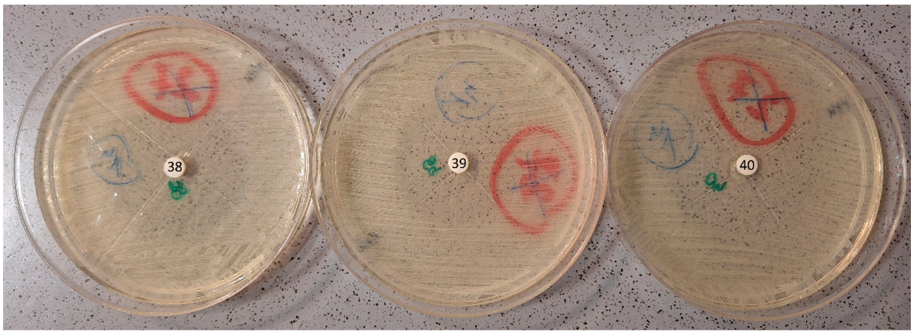

Bacillus cereus ATCC 10876

Hydrophilic NADES with positive-control

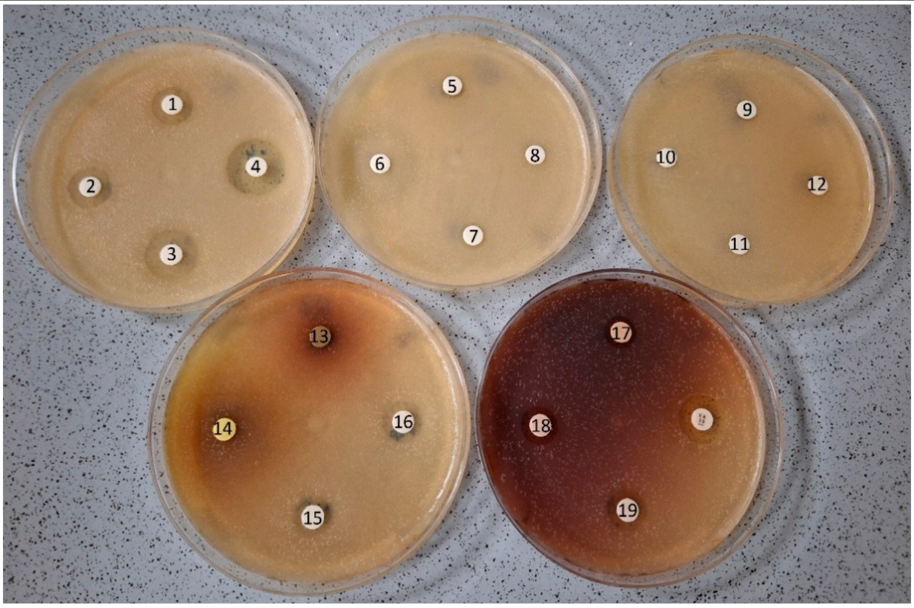

Lipophilic NADES

***1,8*-Cineole:Thymol**

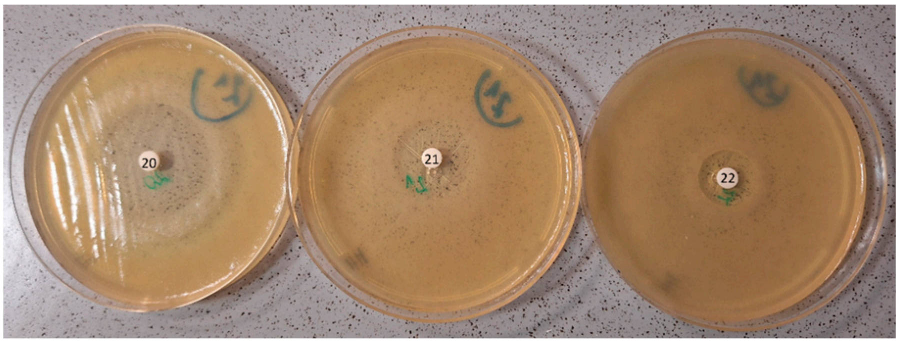

***1,8*-Cineole:DL-Menthol**

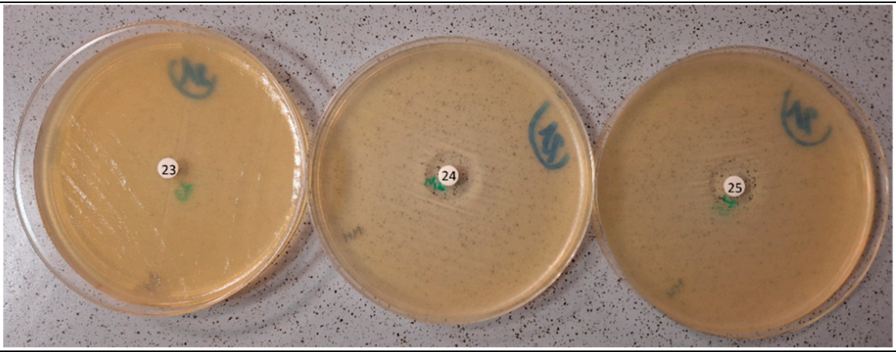

Camphor:Thymol

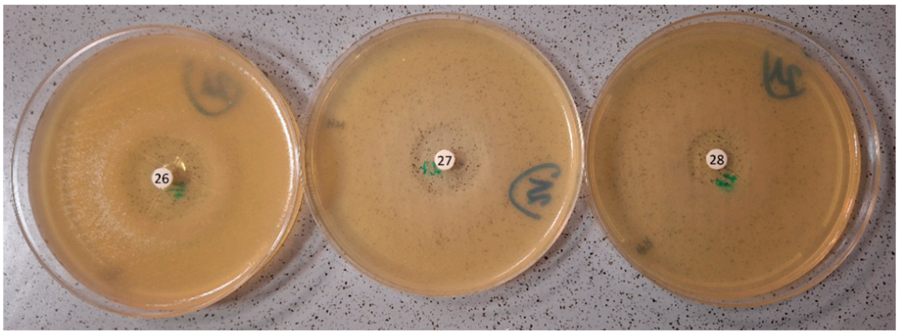

Camphor:DL-Menthol

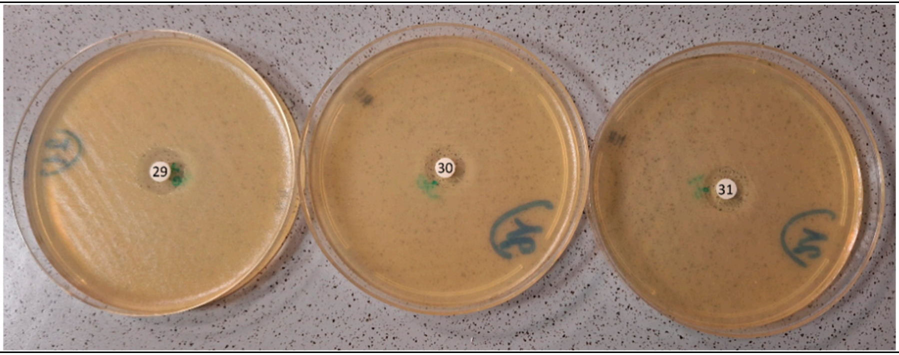

Benzyl alcohol:Thymol

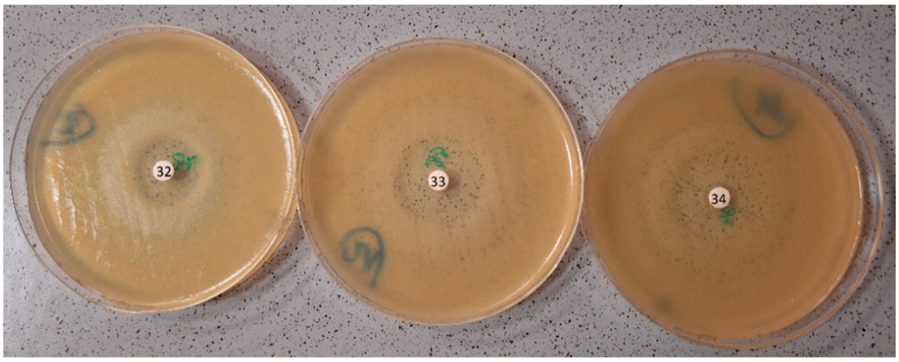

Benzyl alcohol:DL-Menthol

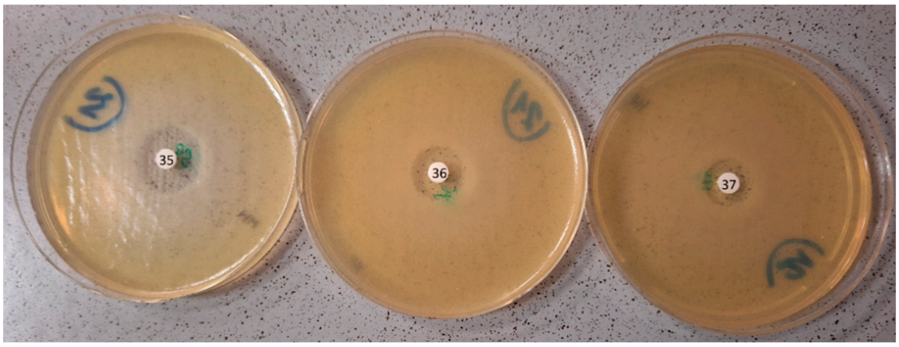

Borneol:Thymol

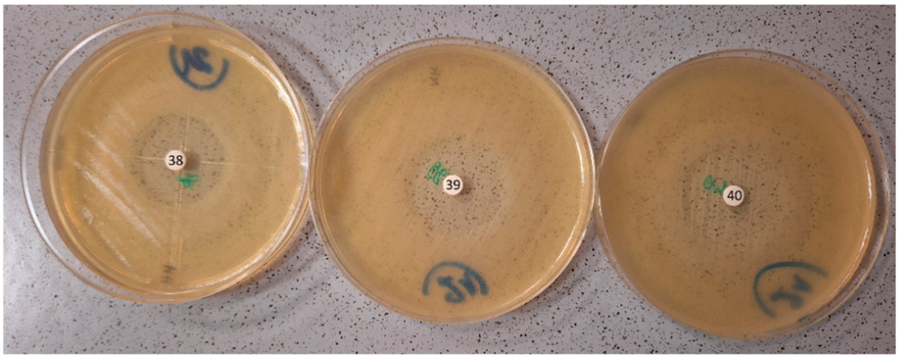

***Staphylococcus aureus* ATCC 29213 (MSSA)**

Hydrophilic NADES with positive-control

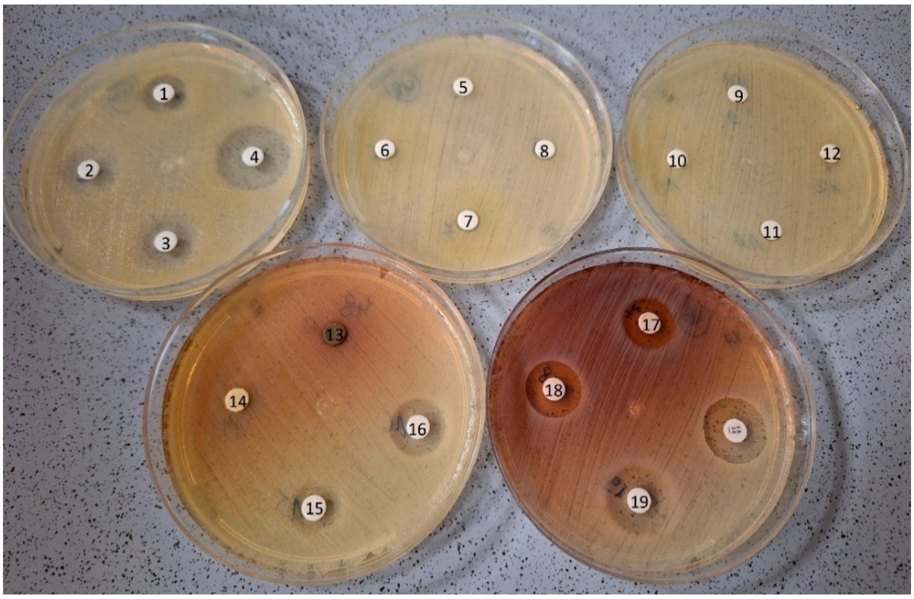

Lipophilic NADES

***1,8*-Cineole:Thymol**

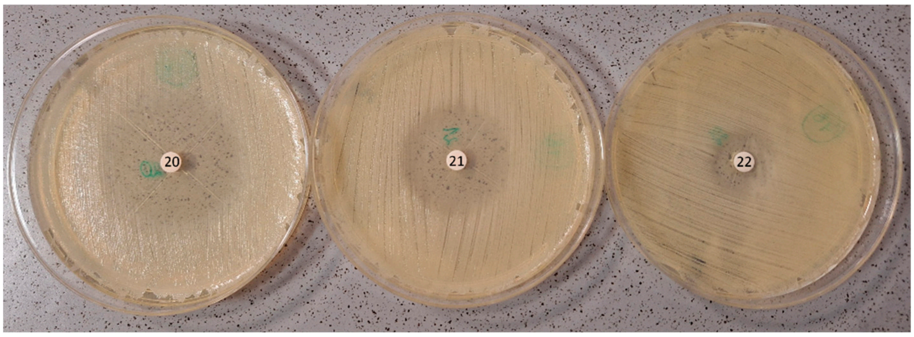

***1,8*-Cineole:DL-Menthol**

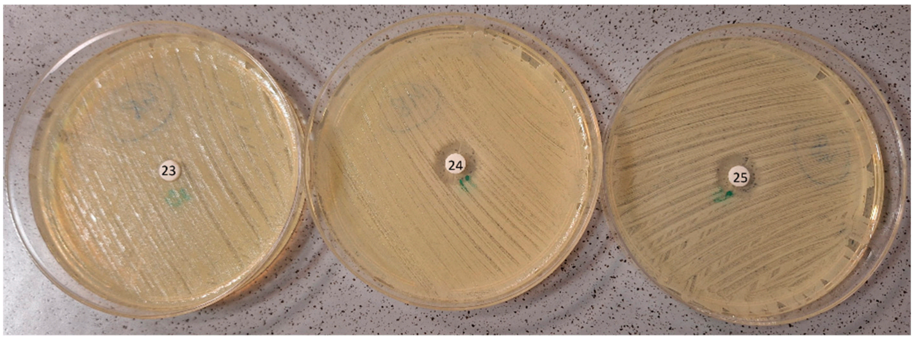

Camphor:Thymol

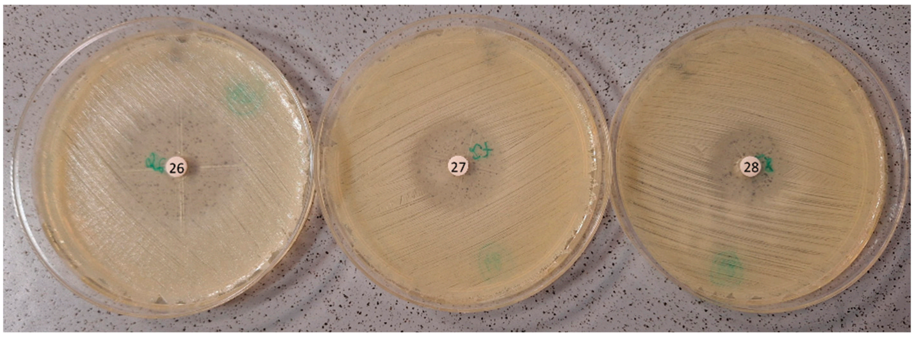

Camphor:DL-Menthol

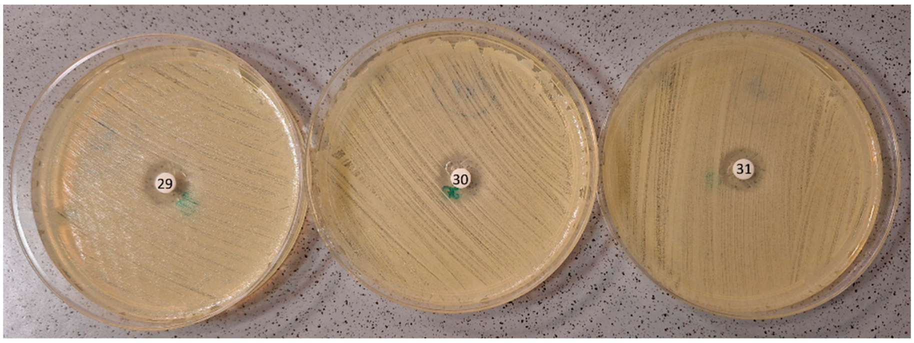

Benzyl alcohol:Thymol

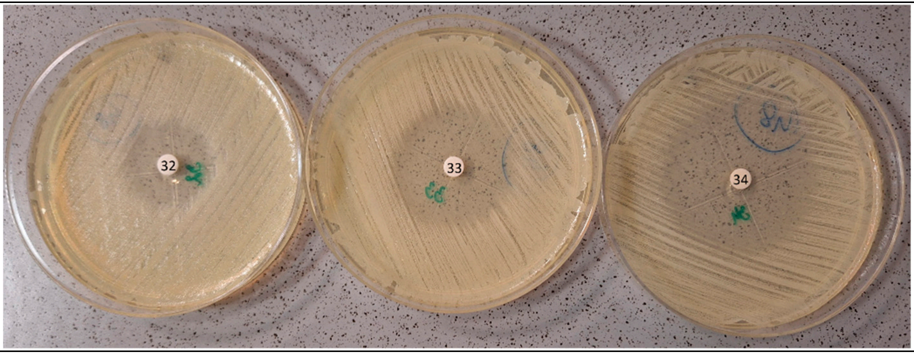

Benzyl alcohol:DL-Menthol

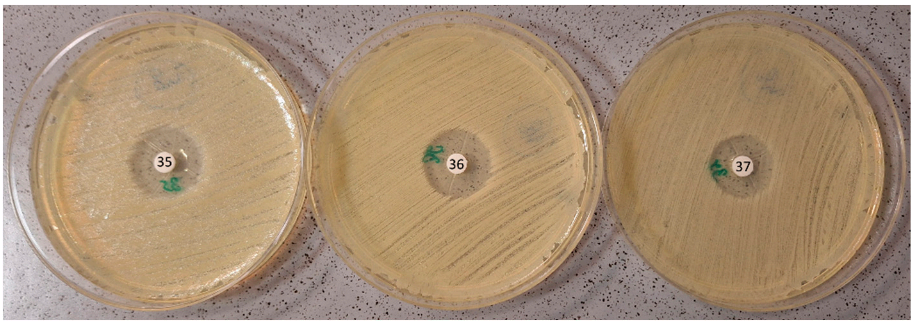

Borneol:Thymol

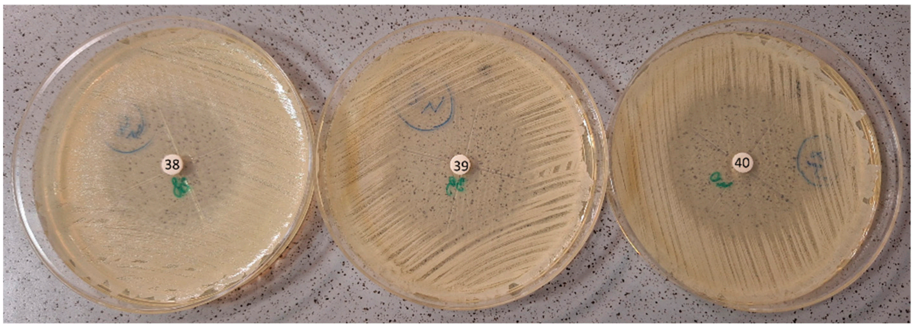

Staphylococcus aureus **ATCC BAA-1707 (MRSA)**

Hydrophilic NADES with positive-control

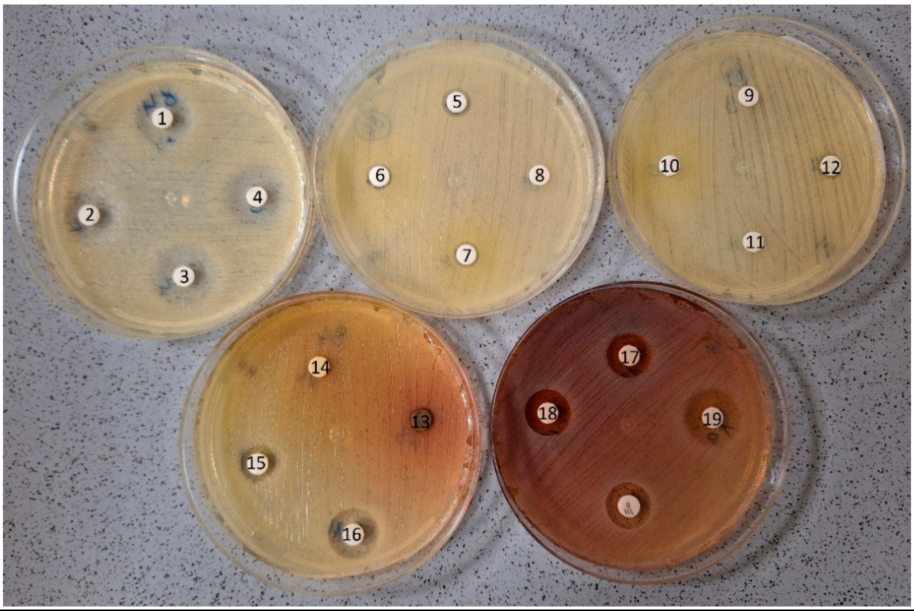

Lipophilic NADES

***1,8*-Cineole:Thymol**

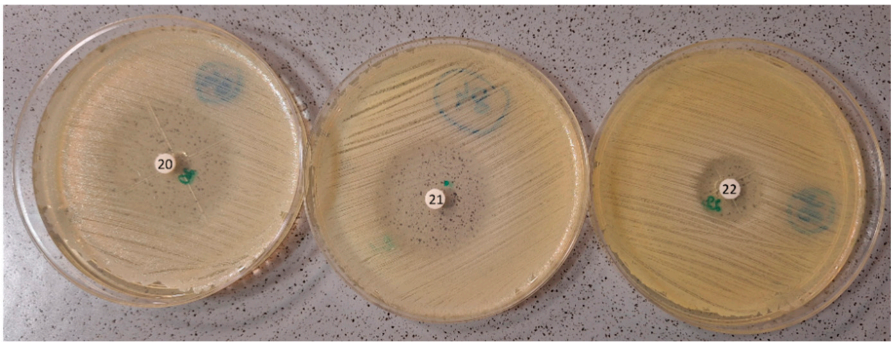

***1,8*-Cineole:DL-Menthol**

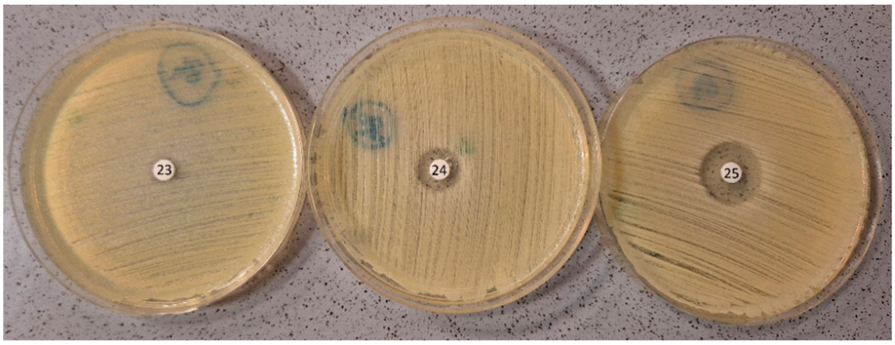

Camphor:Thymol

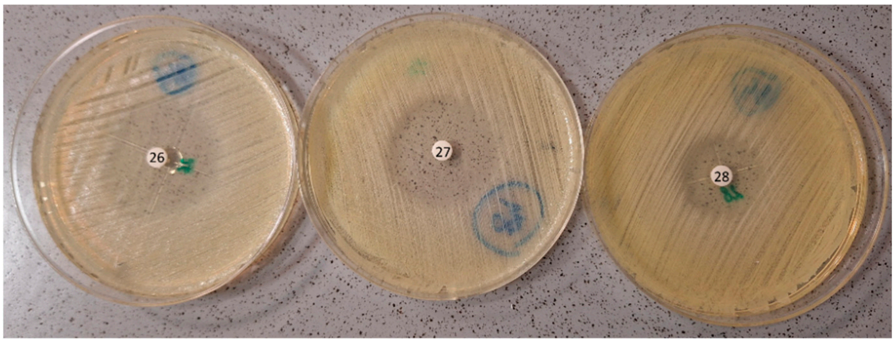

Camphor:DL-Menthol

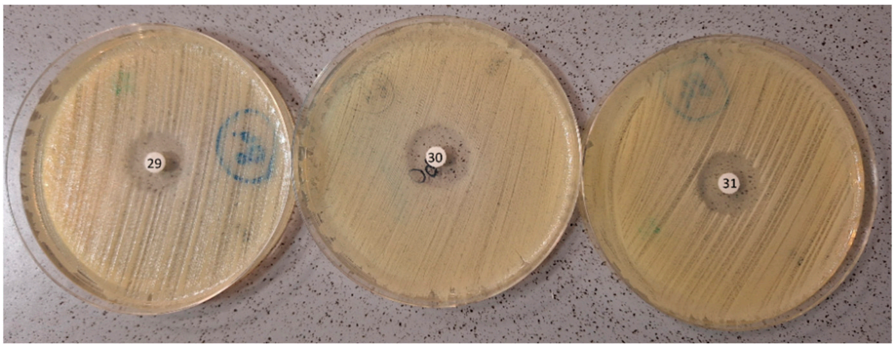

Benzyl alcohol:Thymol

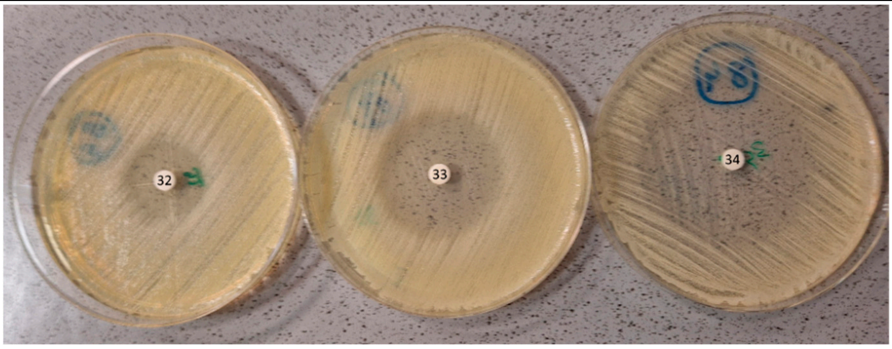

Benzyl Alcohol:DL-Menthol

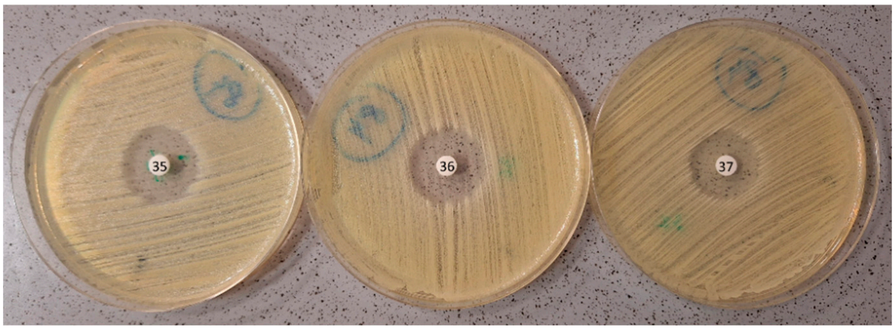

Borneol:Thymol

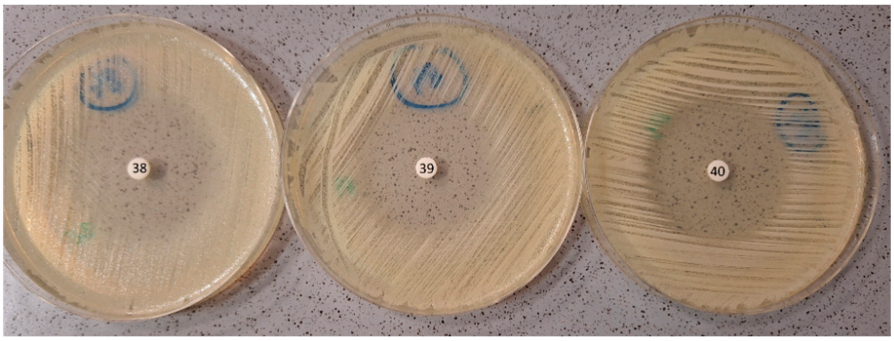

Enterococcus faecalis ATCC 51299

Hydrophilic NADES with positive-control

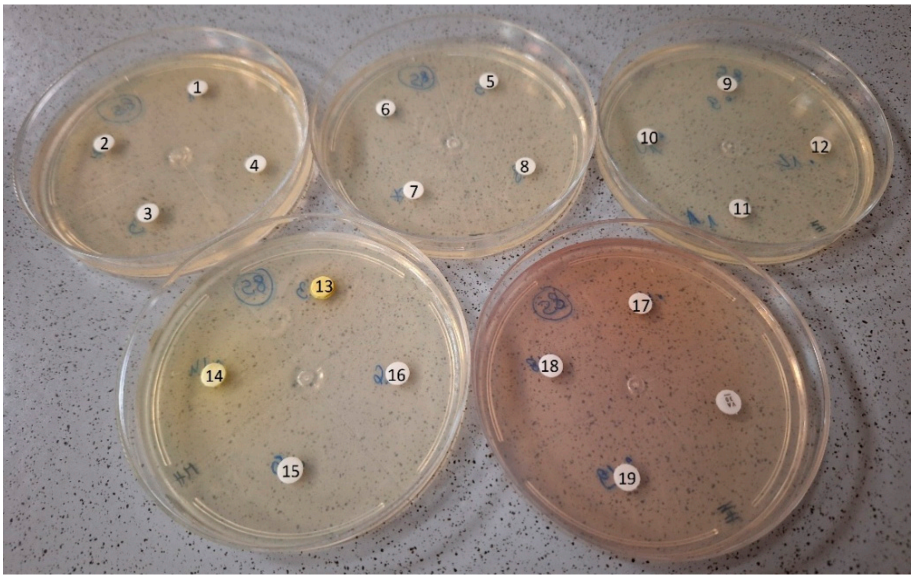

Lipophilic NADES

***1,8*-Cineole:Thymol**

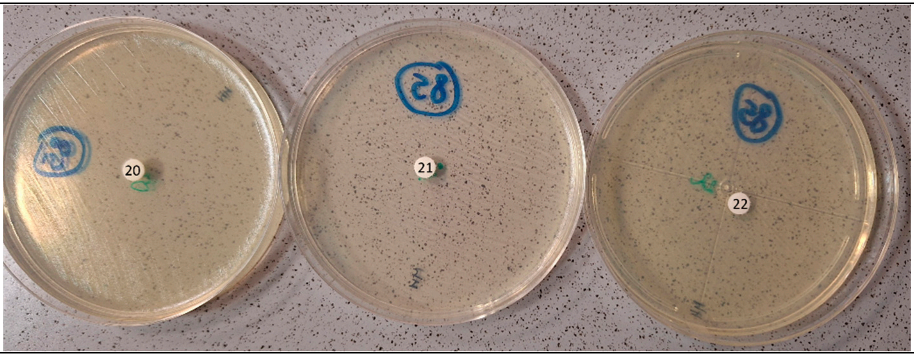

1,8*-Cineole:*DL-Menthol

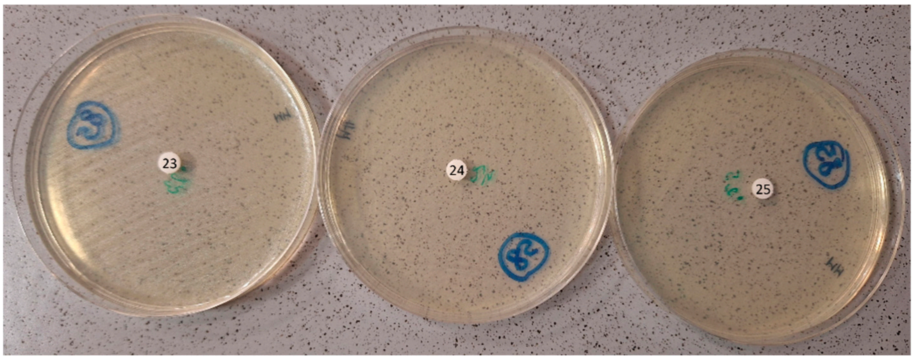

Camphor:Thymol

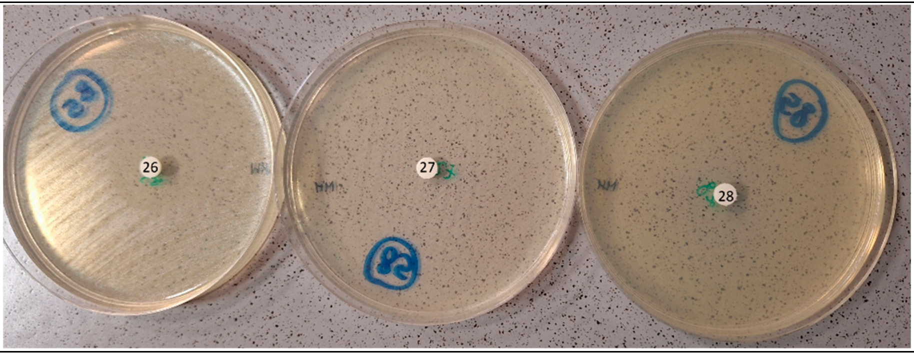

Camphor:DL-Menthol

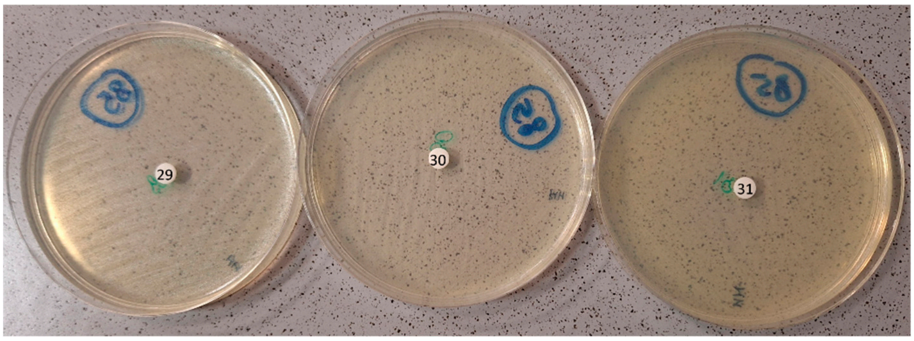

Benzyl alcohol:Thymol

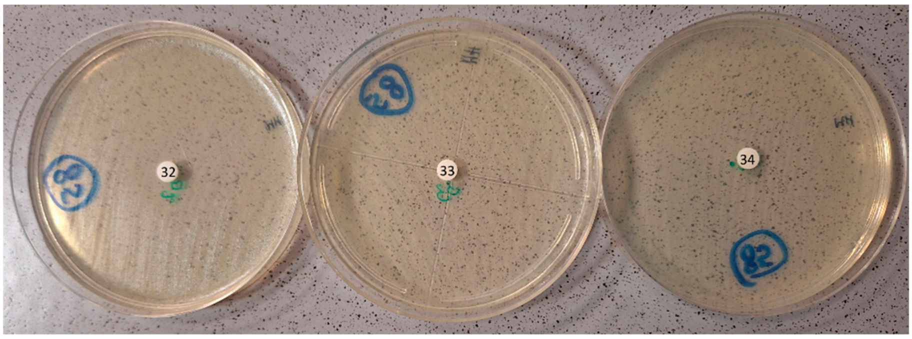

Benzyl alcohol:DL-Menthol

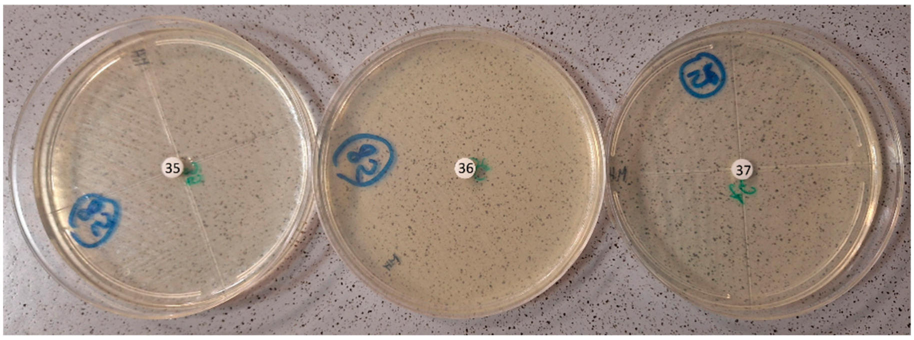

Borneol:Thymol

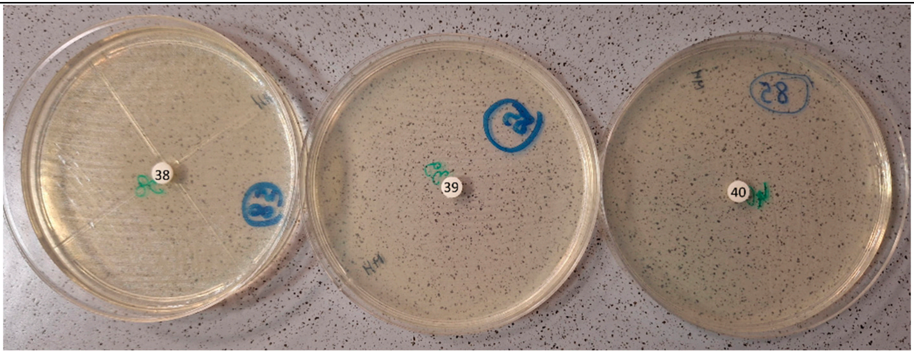

Salmonella Tiphimurium ATCC 14028

Hydrophilic NADES with positive-control

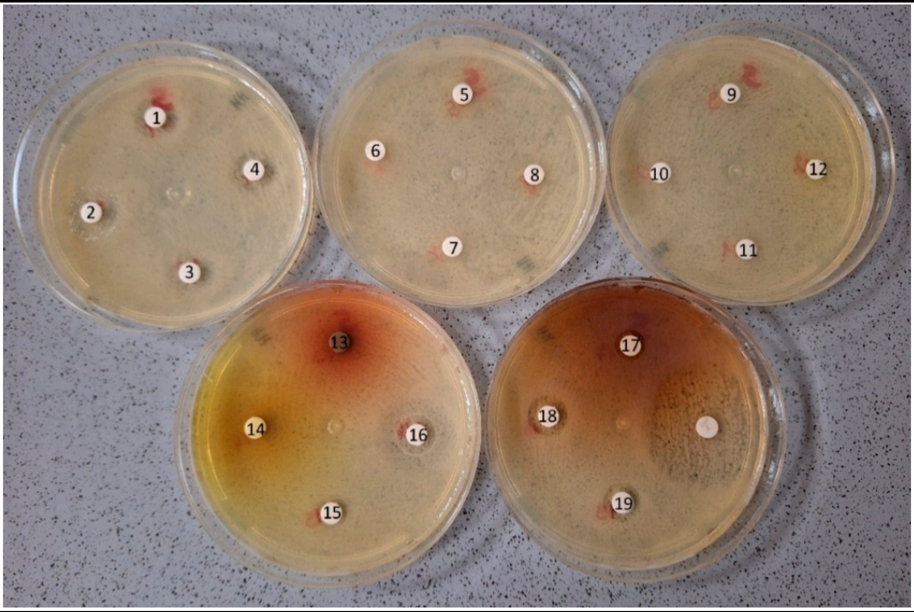

Lipophilic NADES

1,8-Cineole:Thymol

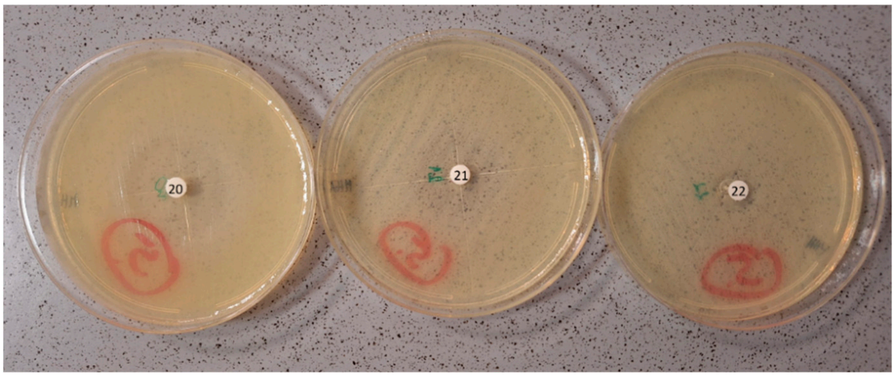

1,8-Cineole:DL-Menthol

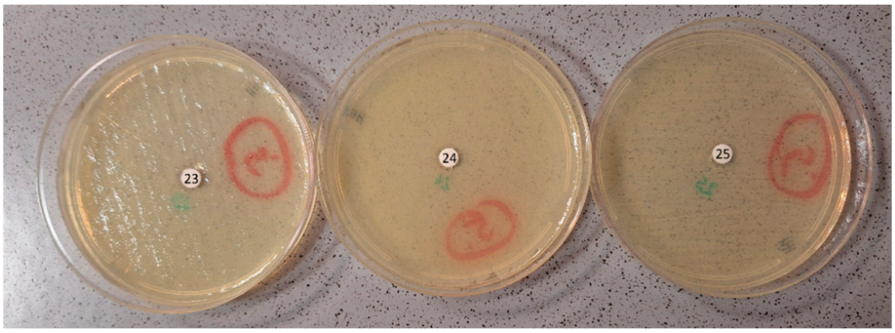

Camphor:Thymol

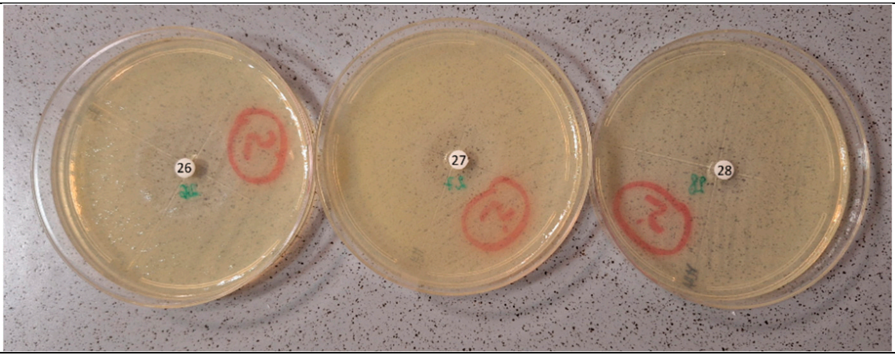

Camphor:DL-Menthol

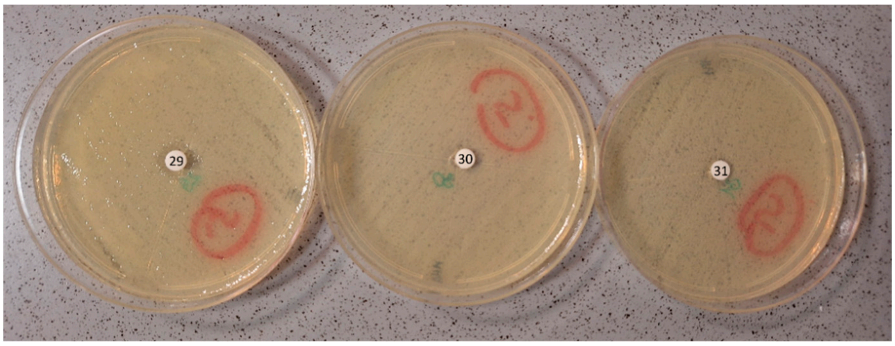

Benzyl alcohol:Thymol

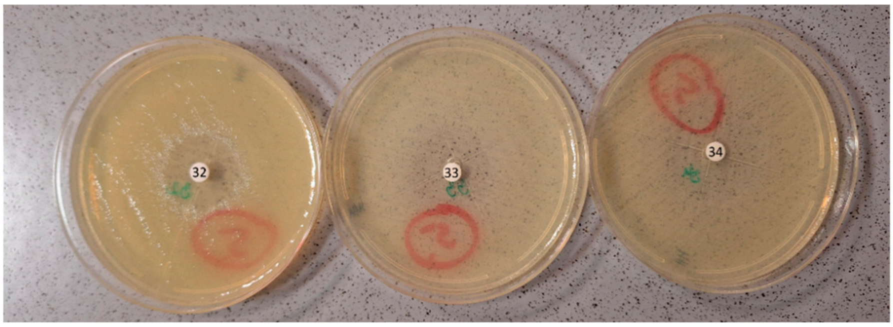

Benzyl alcohol:DL-Menthol

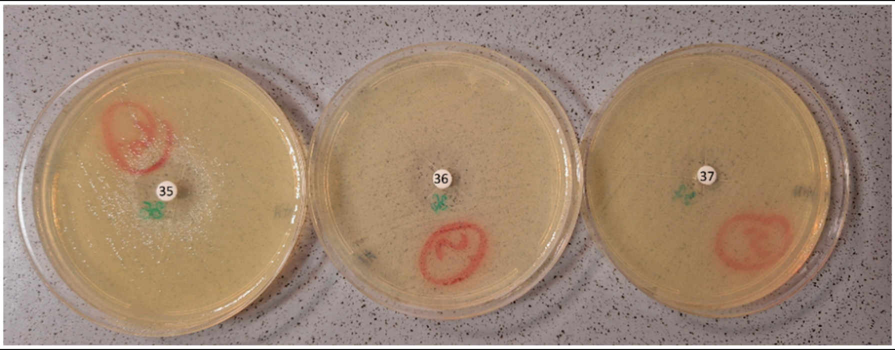

Borneol:Thymol

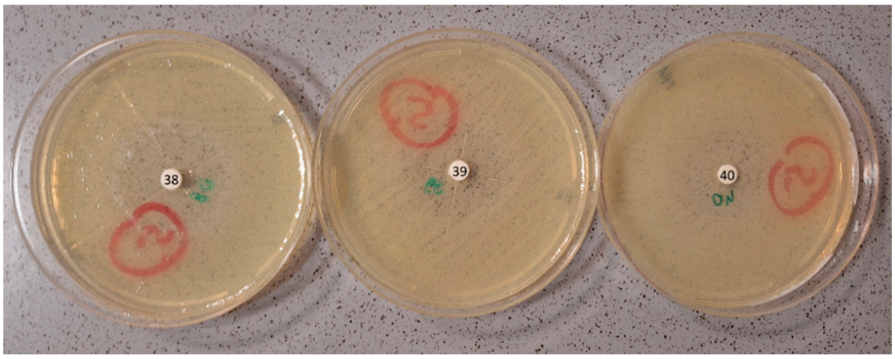

Escherichia coli ATCC 25922

Hydrophilic NADES with positive-control

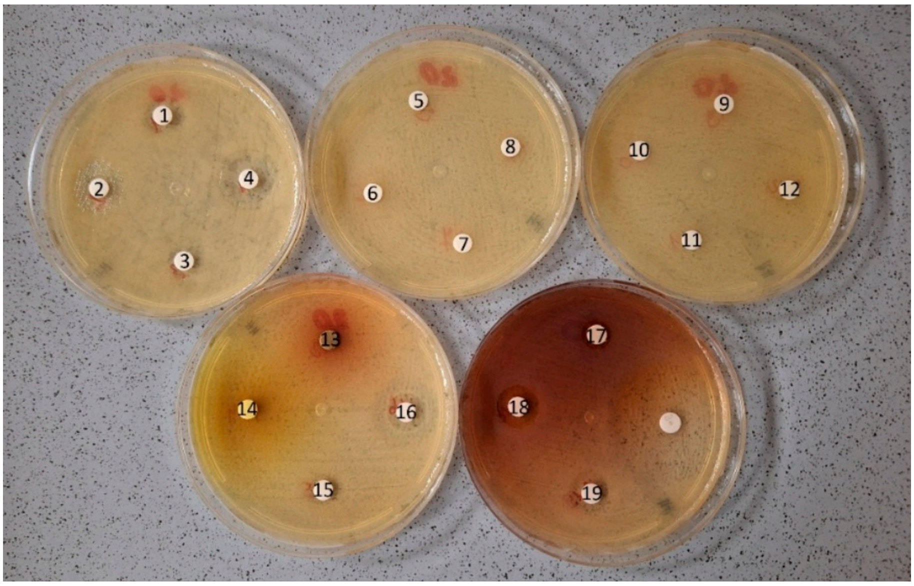

Lipophilic NADES

1,8-Cineole:Thymol

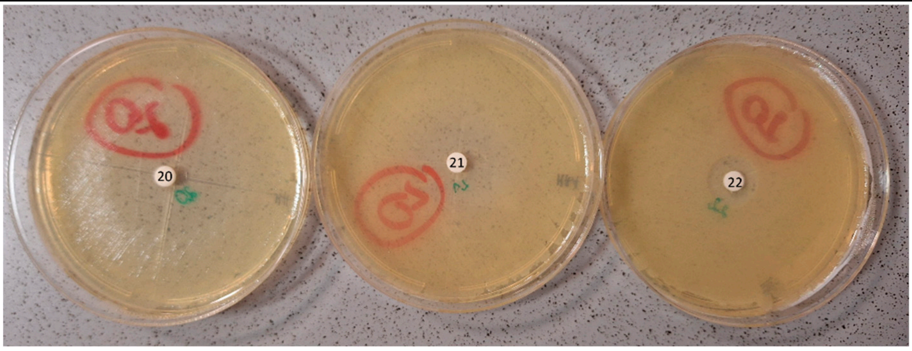

1,8-Cineole:DL-Menthol

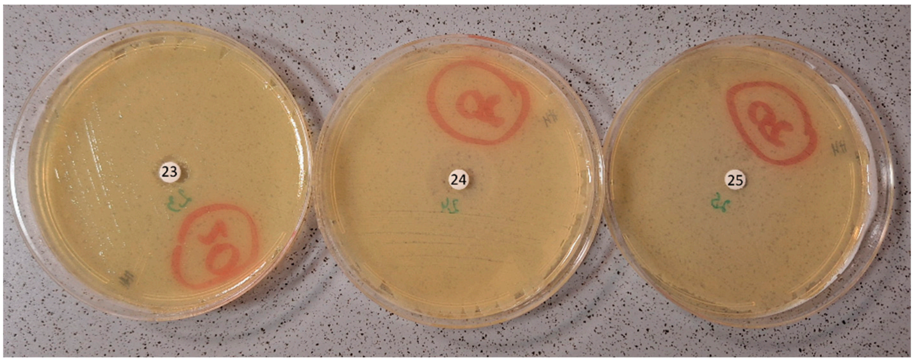

Camphor:Thymol

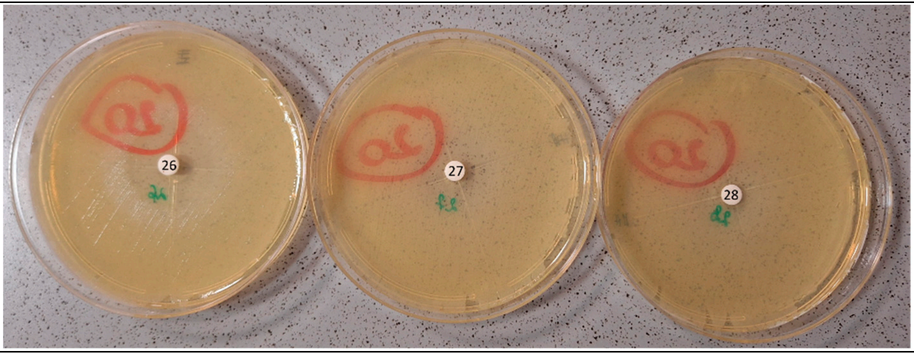

Camphor:DL-Menthol

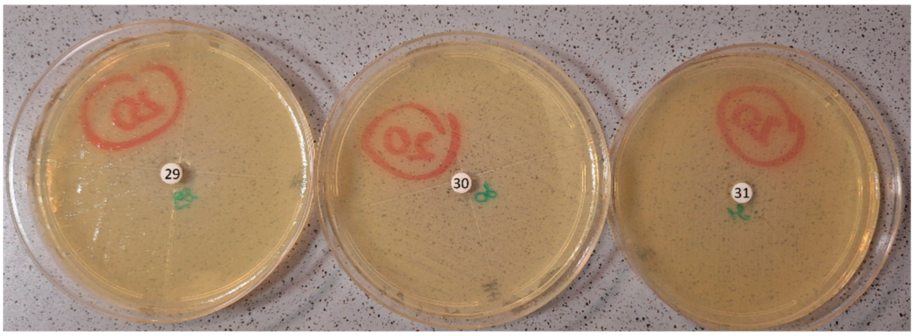

Benzyl alcohol:Thymol

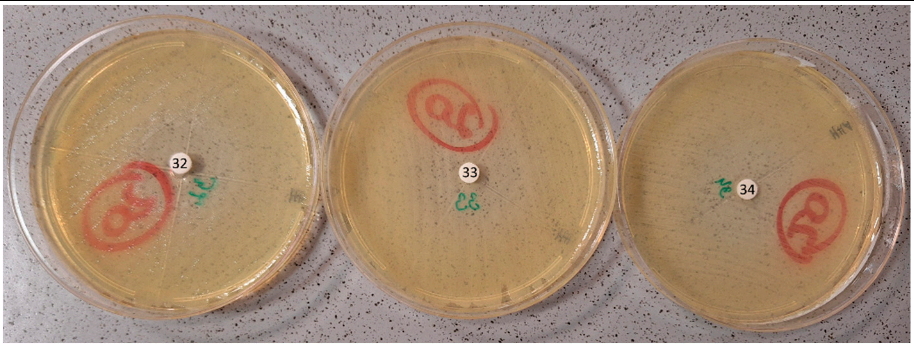

Benzyl alcohol:DL-Menthol

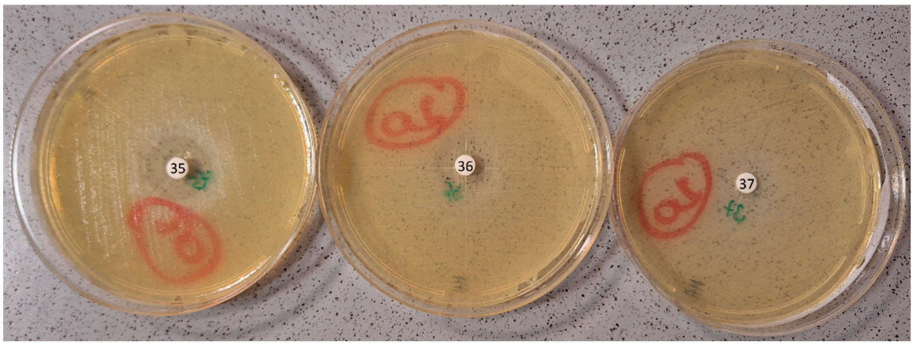

Borneol:Thymol

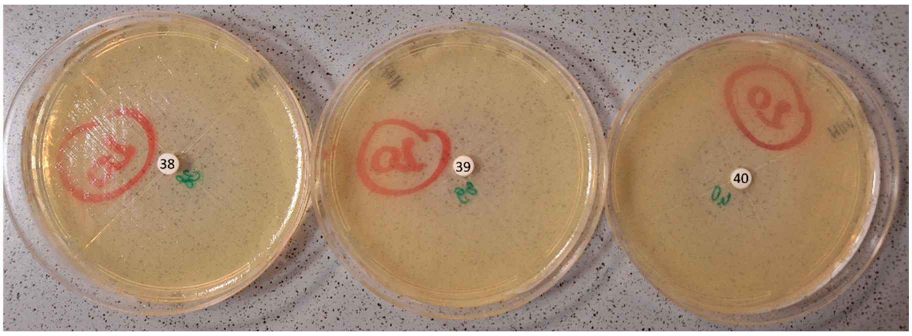

Pseudomonas aeruginosa ATCC 27853

Hydrophilic NADES with positive-control

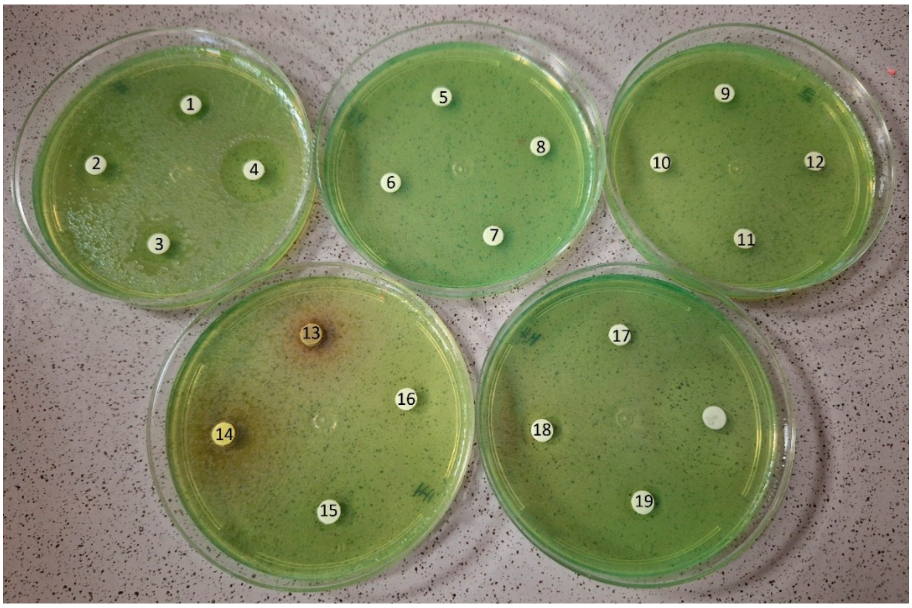

Lipophilic NADES

***1,8*-Cineole:Thymol**

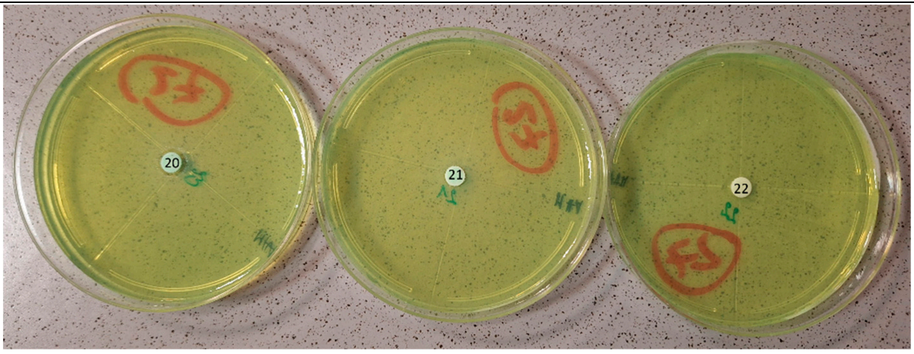

***1,8*-Cineole:DL-Menthol**

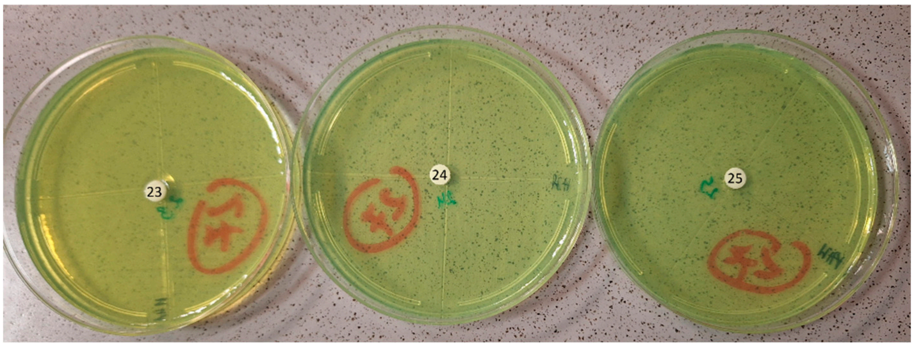

Camphor:Thymol

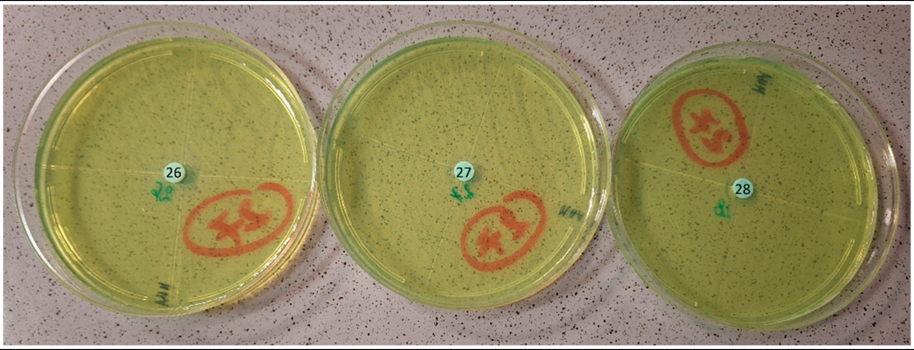

Camphor:DL-Menthol

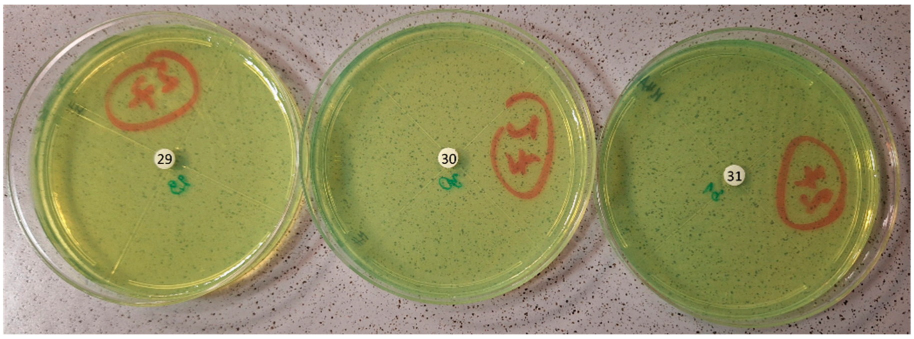

Benzyl alcohol:Thymol

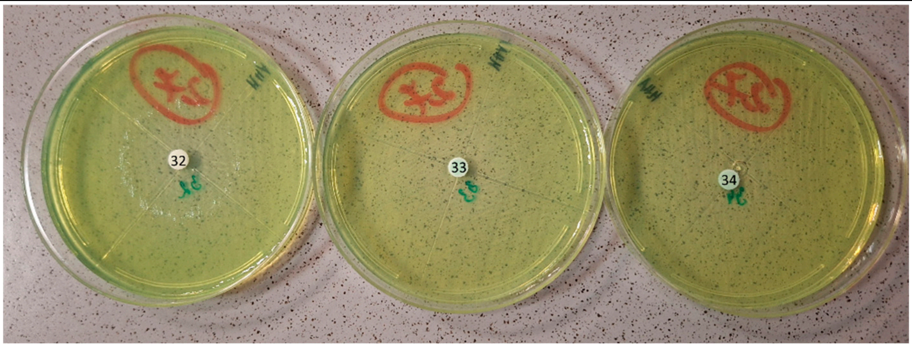

Benzyl alcohol:DL-Menthol

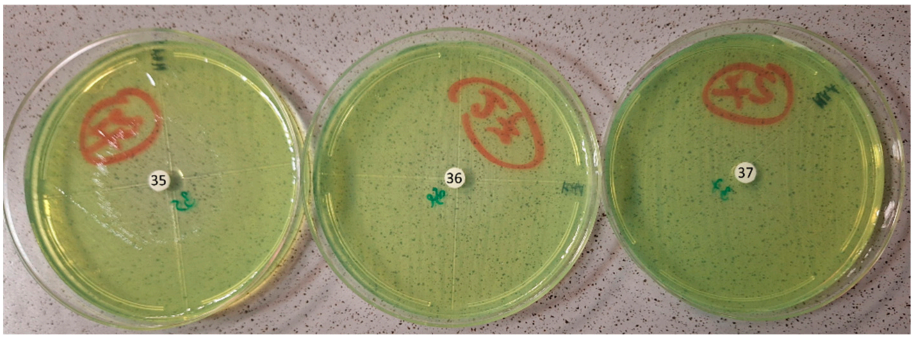

Borneol:Thymol

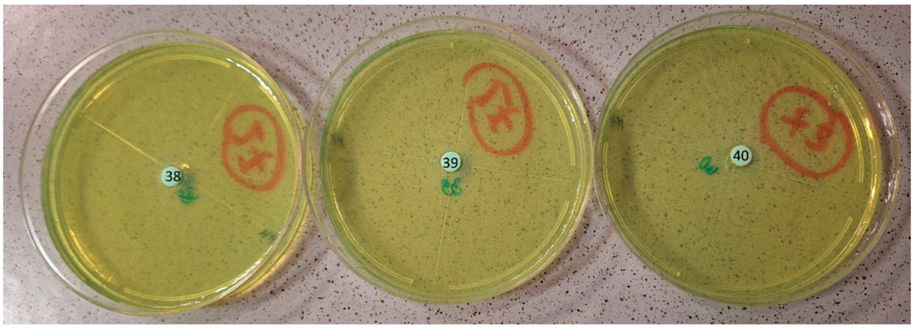

***Candida auris* CDC B11903**

Hydrophilic NADES

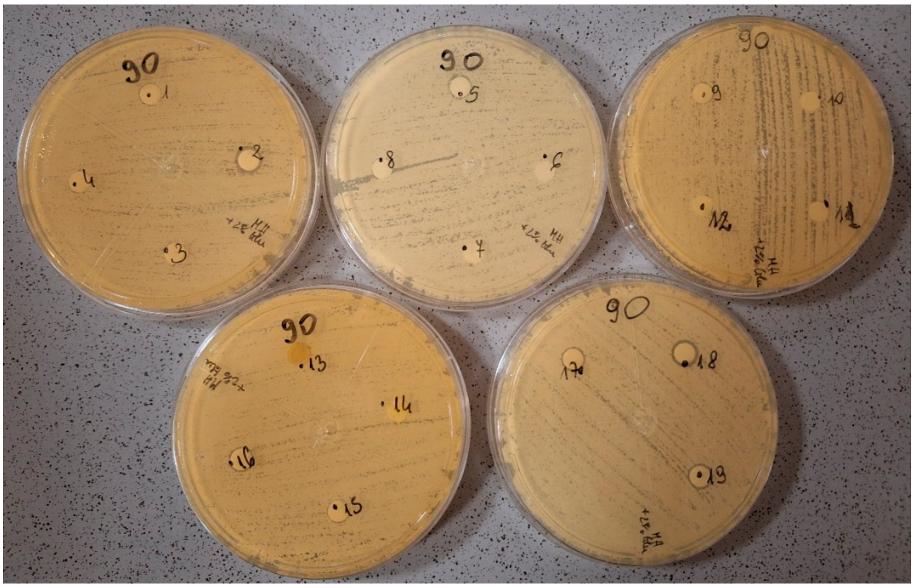

Positive control

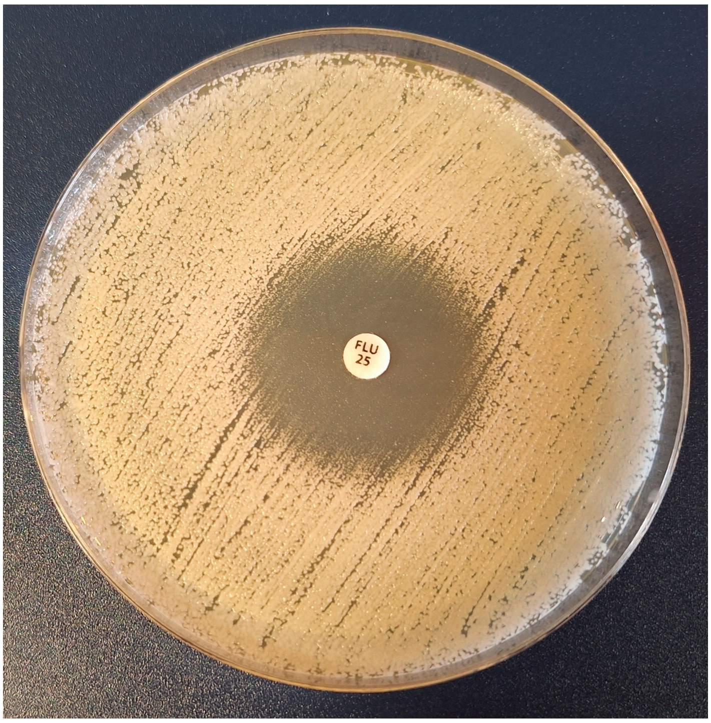

Lipophilic NADES

***1,8*-Cineole:Thymol**

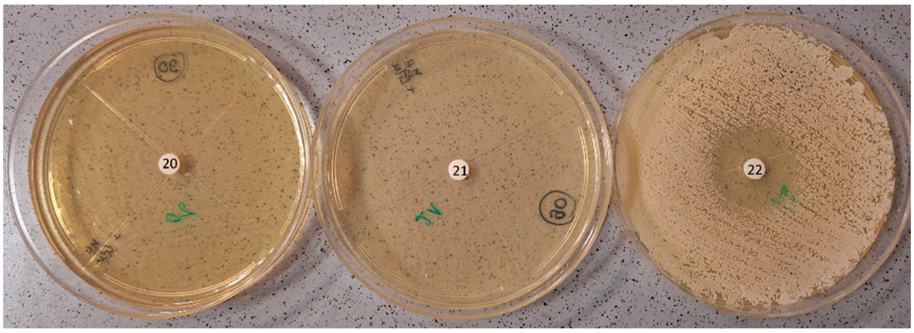

***1,8*-Cineole:DL-Menthol**

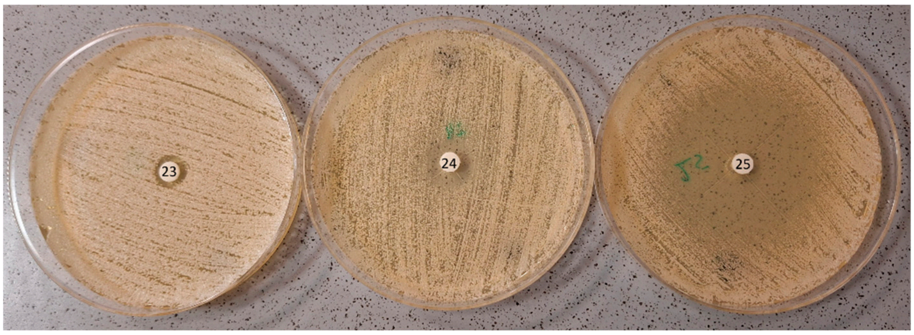

Camphor:Thymol

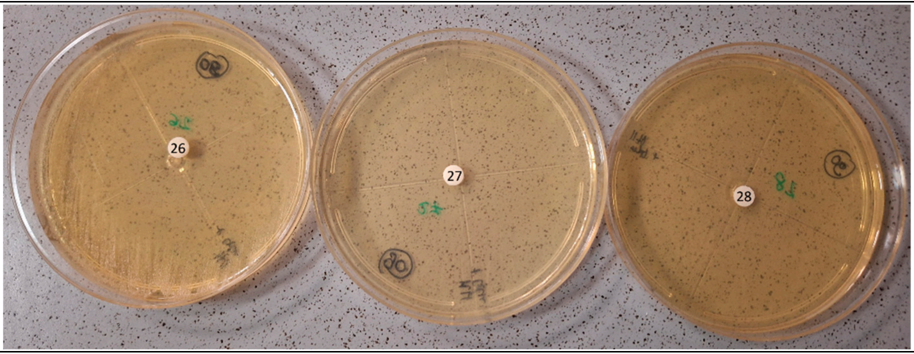

Camphor:DL-Menthol

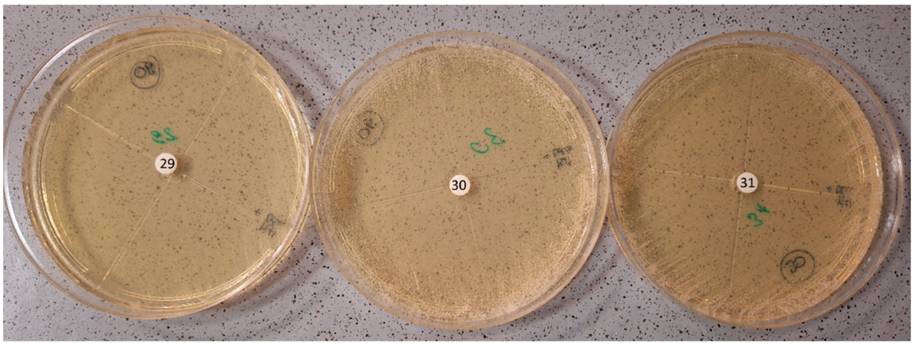

Benzyl alcohol:Thymol

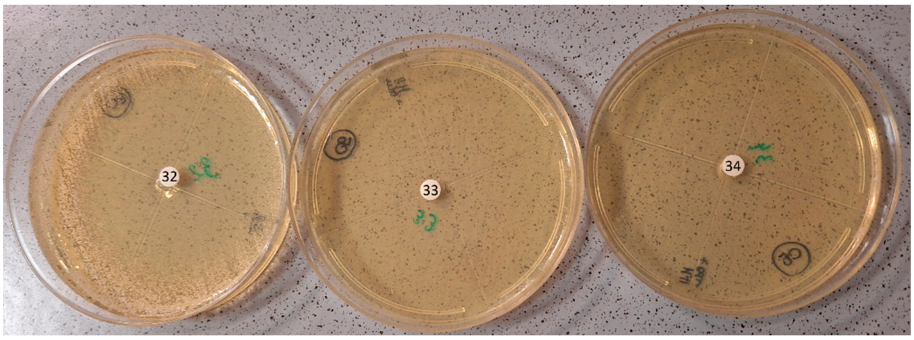

Benzyl alcohol:DL-Menthol

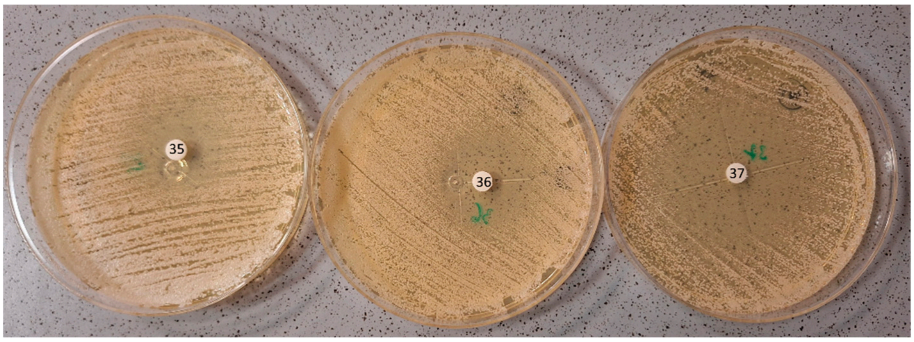

Borneol:Thymol

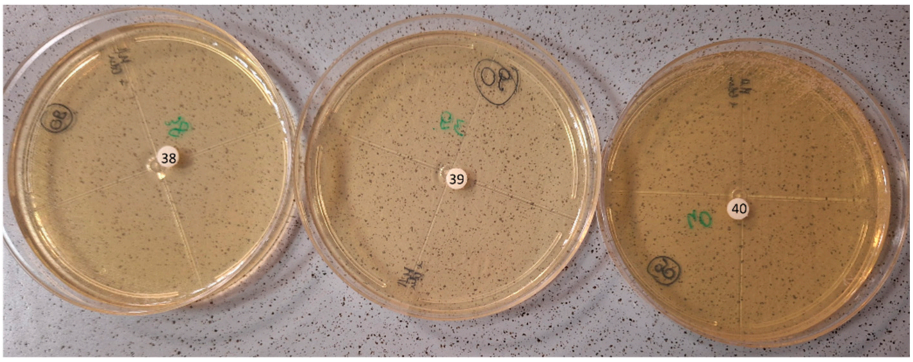

Candida albicans ATCC 10231

Hydrophilic NADES

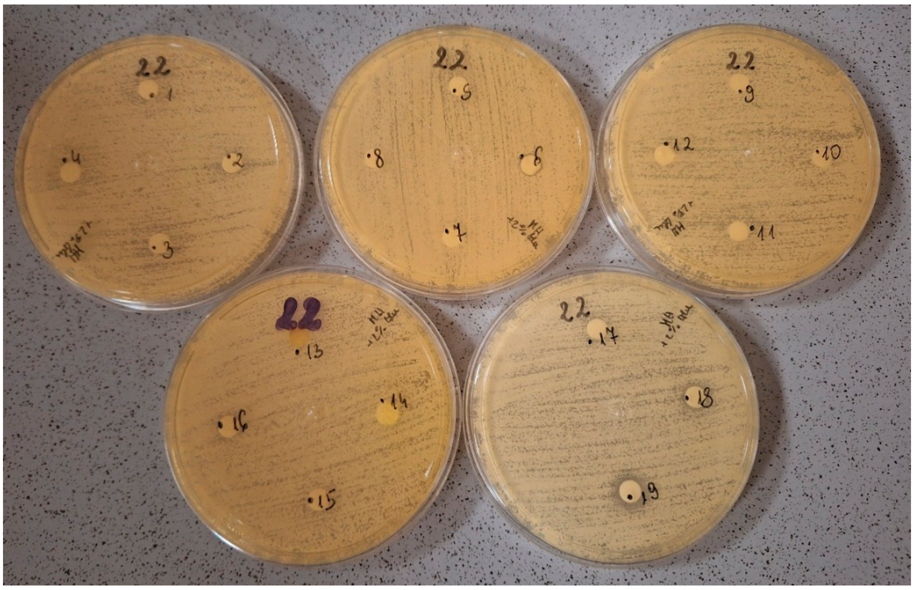

Positive control

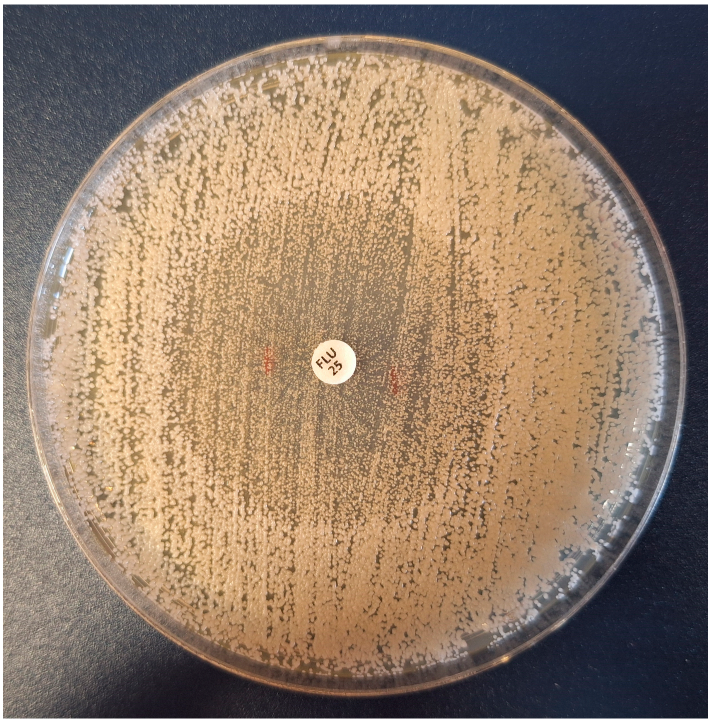

Lipophilic NADES

***1,8*-Cineole:Thymol**

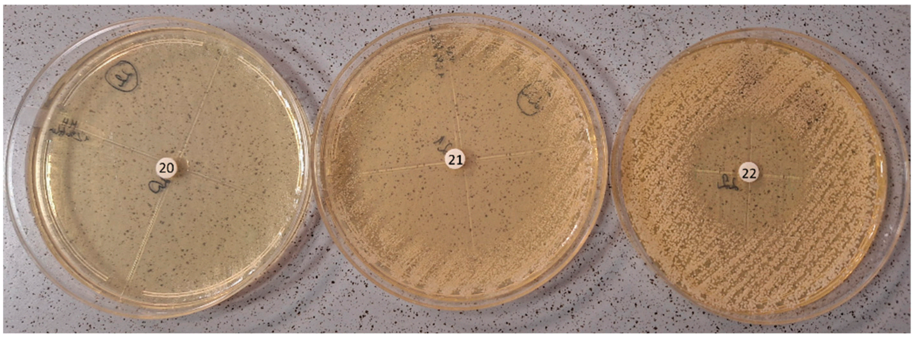

1,8-Cineole:DL-Menthol

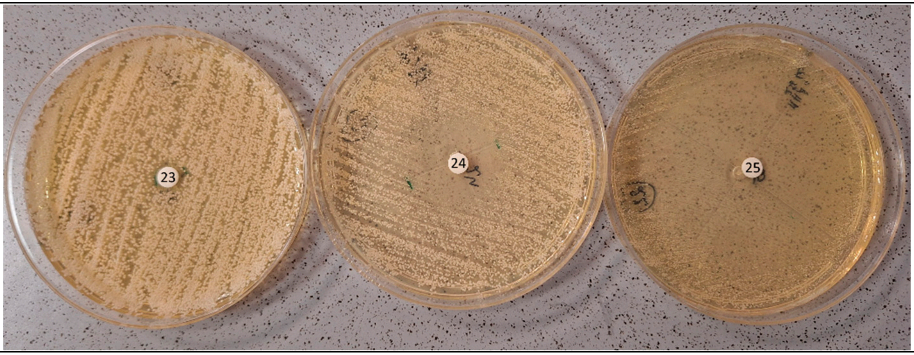

Camphor:Thymol

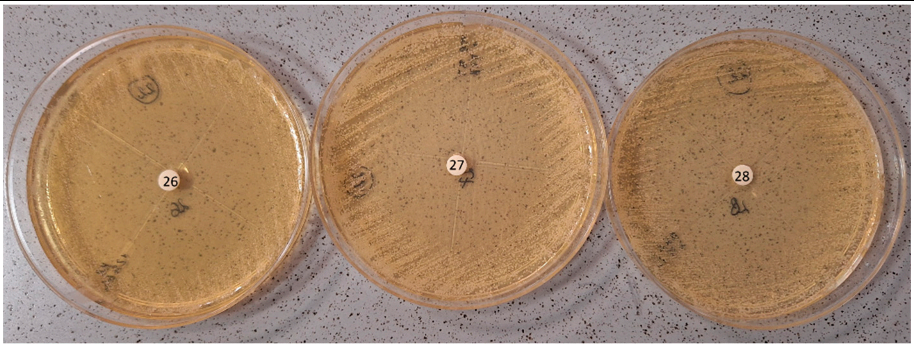

Camphor:DL-Menthol

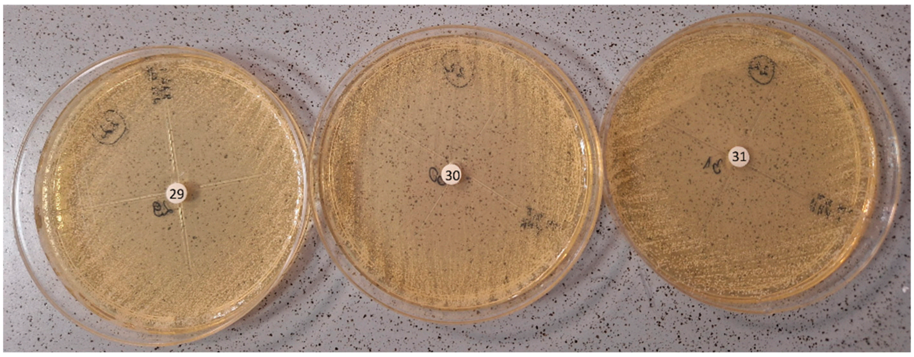

Benzyl alcohol:Thymol

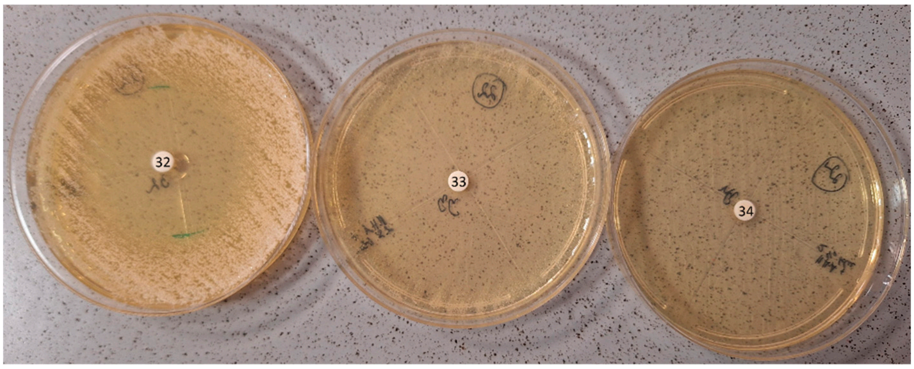

Benzyl alcohol:DL-Menthol

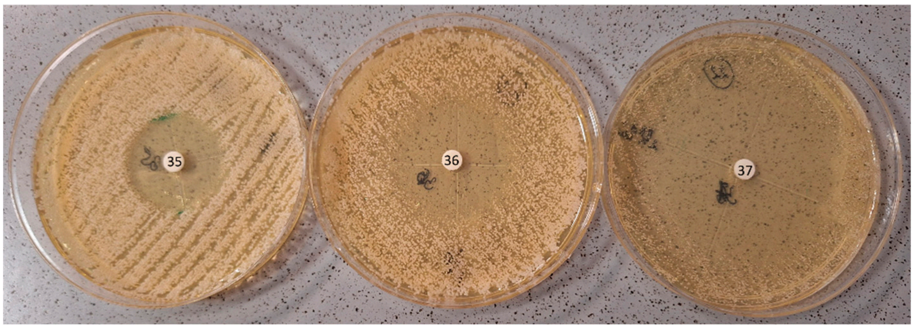

Borneol:Thymol

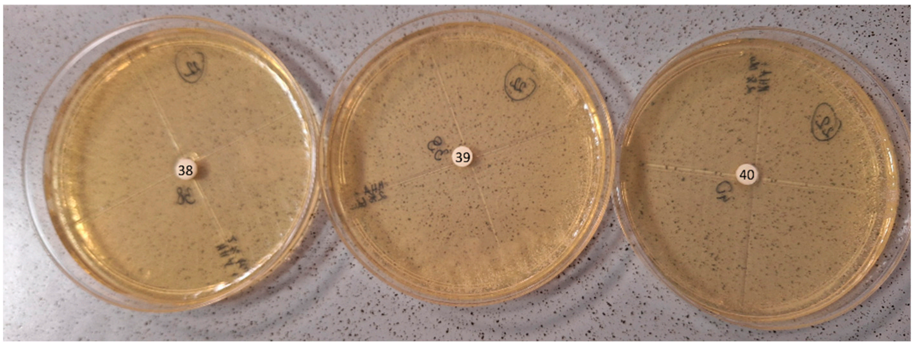

Candida glabrata ATCC 90030

Hydrophilic NADES

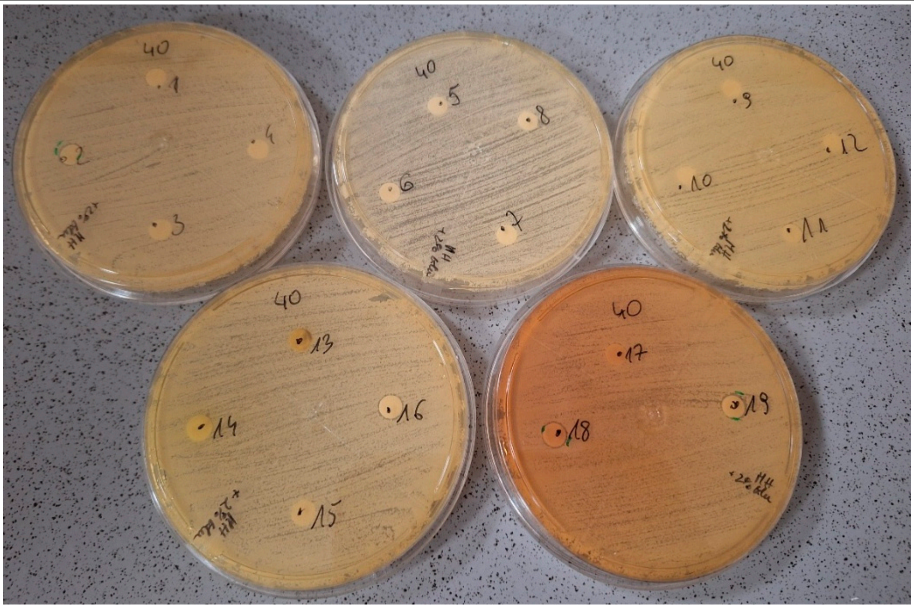

Positive control

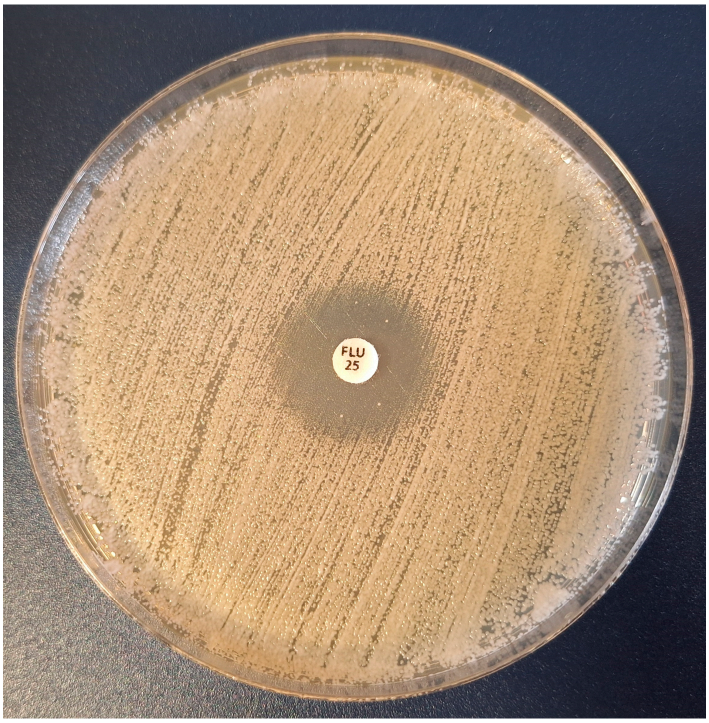

Lipophilic NADES

***1,8*-Cineole:Thymol**

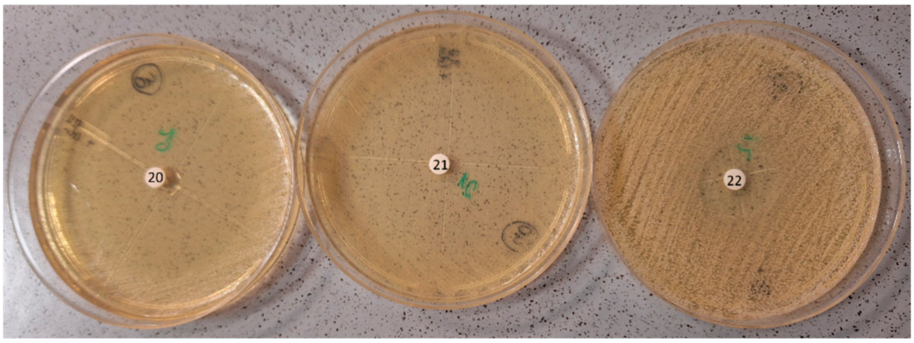

***1,8*-Cineole:DL-Menthol**

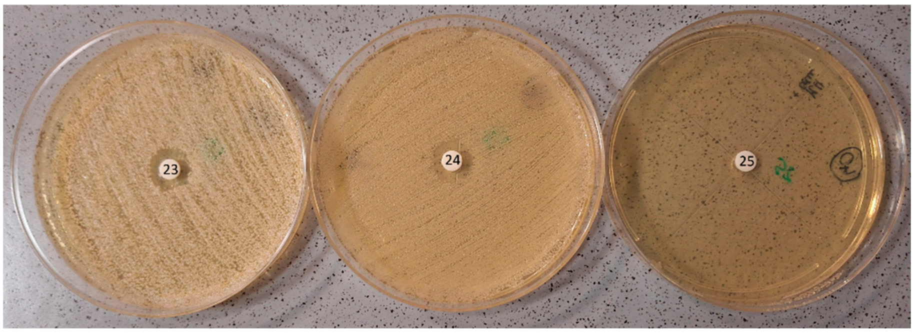

Camphor:Thymol

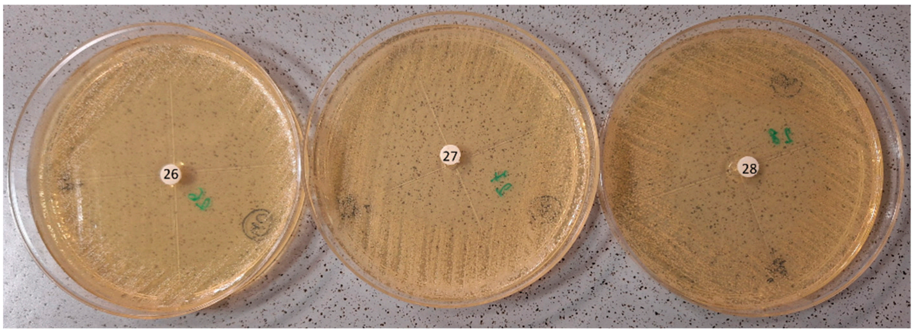

Camphor:DL-Menthol

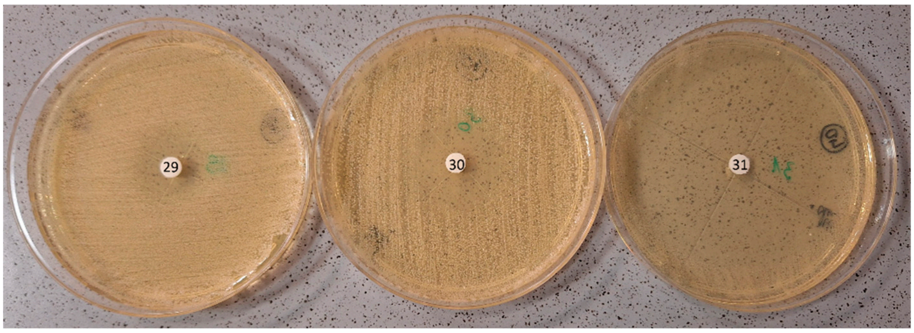

Benzyl alcohol:Thymol

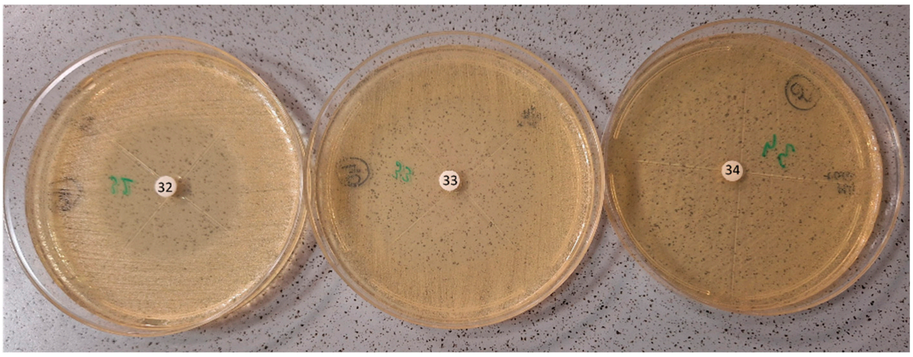

Benzyl alcohol:DL-Menthol

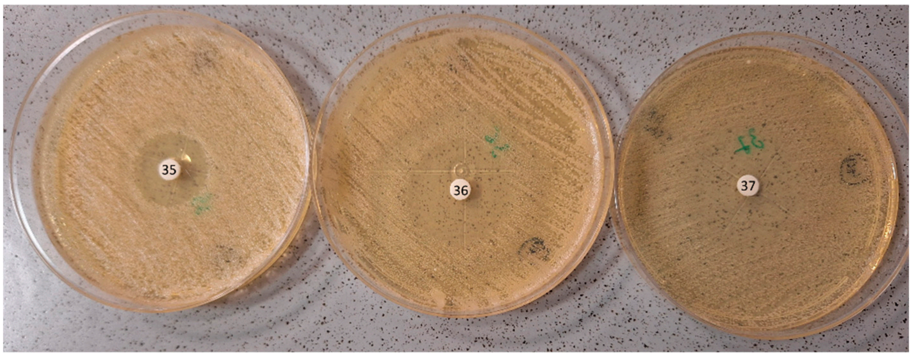

Borneol:Thymol

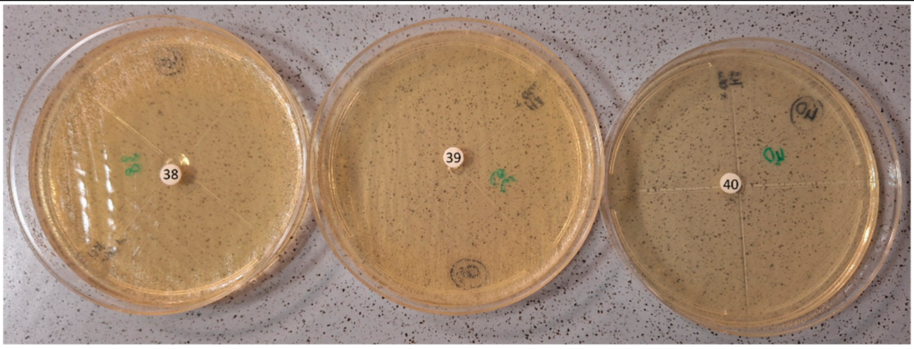

Supplement: Supplementary file 1 [file molecules-30-04219-s001.zip › molecules-3937647-supplementary.pdf]
